# Enhancing dysarthric speech recognition through SepFormer and hierarchical attention network models with multistage transfer learning

**DOI:** 10.1038/s41598-024-80764-w

**Published:** 2024-11-27

**Authors:** R. Vinotha, D. Hepsiba, L. D. Vijay Anand, J. Andrew, R. Jennifer Eunice

**Affiliations:** 1 Division of Robotics Engineering, Karunya Institute of Technology and Sciences, Coimbatore, Tamil Nadu India; 2 Division of Biomedical Engineering, Karunya Institute of Technology and Sciences, Coimbatore, Tamil Nadu India; 3 Department of Computer Science and Engineering, Manipal Institute of Technology, Manipal Academy of Higher Education, Manipal, Karnataka India; 4 Department of Mechatronics Engineering, Manipal Institute of Technology, Manipal Academy of Higher Education, Manipal, Karnataka India

**Keywords:** SepFormer-SEGAN, HAN, Transformer, Conformer, DSR, Dysarthric speech enhancement, WRA, Computational biology and bioinformatics, Computational models

## Abstract

Dysarthria, a motor speech disorder that impacts articulation and speech clarity, presents significant challenges for Automatic Speech Recognition (ASR) systems. This study proposes a groundbreaking approach to enhance the accuracy of Dysarthric Speech Recognition (DSR). A primary innovation lies in the integration of the SepFormer-Speech Enhancement Generative Adversarial Network (S-SEGAN), an advanced generative adversarial network tailored for Dysarthric Speech Enhancement (DSE), as a front-end processing stage for DSR systems. The S-SEGAN integrates SEGAN’s adversarial learning with SepFormer speech separation capabilities, demonstrating significant improvements in performance. Furthermore, a multistage transfer learning approach is employed to assess the DSR models for both word-level and sentence-level DSR. These DSR models are first trained on a large speech dataset (LibriSpeech) and then fine-tuned on dysarthric speech data (both isolated and augmented). Evaluations demonstrate significant DSR accuracy improvements in DSE integration. The Dysarthric Speech (DS)-baseline models (without DSE), Transformer and Conformer achieved Word Recognition Accuracy (WRA) percentages of 68.60% and 69.87%, respectively. The introduction of Hierarchical Attention Network (HAN) with the Transformer and Conformer architectures resulted in improved performance, with T-HAN achieving a WRA of 71.07% and C-HAN reaching 73%. The Transformer model with DSE + DSR for isolated words achieves a WRA of 73.40%, while that of the Conformer model reaches 74.33%. Notably, the T-HAN and C-HAN models with DSE + DSR demonstrate even more substantial enhancements, with WRAs of 75.73% and 76.87%, respectively. Augmenting words further boosts model performance, with the Transformer and Conformer models achieving WRAs of 76.47% and 79.20%, respectively. Remarkably, the T-HAN and C-HAN models with DSE + DSR and augmented words exhibit WRAs of 82.13% and 84.07%, respectively, with C-HAN displaying the highest performance among all proposed models.

## Introduction

Clear communication is essential for human interaction, but individuals with dysarthria, a motor speech disorder, face significant challenges. Dysarthric speech is characterized by slurred words, imprecise pronunciation, and reduced volume, complicating communication for both speakers and listeners. This disorder arises from disruptions between the brain and speech muscles, leading to impaired muscle control and articulation. The American Speech-Language-Hearing Association (ASHA) estimates that dysarthria affects approximately 1 in 100 people globally. The National Institute on Deafness and Other Communication Disorders (NIDCD) notes that the prevalence in elderly individuals ranges from 1 to 10%, depending on diagnostic criteria. These communication challenges can be further amplified in a world that increasingly relies on ASR technology. ASR has become a crucial tool in various applications, from smartphones to smart home systems, serving as automated assistants that accurately transcribe spoken words^1,2^. ASR fosters effortless multitasking, allowing us to capture ideas or send messages on the go, becoming integral to daily life. Artificial intelligence (AI) has empowered computers to perform tasks traditionally managed by humans, revolutionizing various industries. Notable application include finger vein recognition^3^, diabetic retinopathy detection^4–7^. In recent years, AI’s impact has been transformative across multiple sectors, revolutionizing fields beyond speech recognition^8–11^. In healthcare, AI-powered diagnostic tools and predictive analytics are enhancing patient care and treatment outcomes by enabling early detection of diseases and personalized medicine^12–17^. However, individuals with dysarthria face significant barriers, as their slurred speech often confounds ASR systems. This gap highlights the need for innovative solutions to address their unique communication challenges. The development of ASR systems for dysarthric speech is difficult due to limited data availability, challenges in capturing speech nuances, and distorted spectral features. These issues underscore the necessity for tailored ASR technologies to improve communication for those with speech impairments. Additionally, dysarthric speech is frequently impacted by background noise, further complicating ASR algorithms’ ability to recognize it accurately^18–20^. These factors contribute to the difficulty of recognizing dysarthric speech and limit the development of robust ASR solutions for this population.

Fortunately, the field of deep learning is offering promising solutions to bridge these communication gaps^21–23^. Deep learning algorithms are adept at identifying complex patterns in speech data. This allows them to better handle variations in speech, including accents and background noise. Compared with more traditional approaches, deep learning-based methods have shown significant improvements in speech enhancement performance^24–26^. A category of generative techniques based on Generative Adversarial Network (GAN) has been shown to be effective for speech enhancement, as evidenced by various studies^27–30^. A major hurdle in DSE is capturing the underlying clean speech hidden beneath the distorted spectral features. By leveraging adversarial training, SEGANs can effectively remove noise and artifacts from speech signals, leading to enhanced intelligibility and listener experience^31^. However, their reliance on learning spectral domain representations can prove to be a significant hurdle when dealing with dysarthric speech. Dysarthria, a motor speech disorder, often manifests as distorted spectral features that can hinder the ability of SEGANs to accurately distinguish between noise and the underlying clean speech. The realm of speech enhancement and recognition is witnessing a revolution with the emergence of transformers, a neural network architecture. Unlike traditional methods that struggle to analyze relationships between sounds far apart in a sequence, transformers excel at this task. They can effectively analyze how past sounds influence present sounds, leading to significantly more accurate speech recognition^32^. Within the transformer family, a specific architecture called the conformer^33,34^ is making significant strides in speech recognition. Conformers are more efficient in speech recognition due to their ability to focus on both local and global dependencies, enhance feature extraction, and maintain efficiency with limited data. Speech enhancement is crucial for improving ASR performance in individuals with dysarthria. Although techniques such as the SEGAN have shown promising results, they struggle to accurately reconstruct the underlying clean speech from heavily distorted dysarthric speech. This paper introduces SepFormer-SEGAN, a novel speech enhancement technique that builds upon the SEGAN. The motivation for using SepFormer-SEGAN lies in its dual-stage approach: SepFormer effectively separates speech signals, while the SEGAN framework enhances speech quality by reducing noise and distortions. This combination addresses the high variability and noise in dysarthric speech, producing clearer and more intelligible speech. By improving speech clarity and intelligibility, SepFormer-SEGAN reduces Word Error Rate (WER) and enhances ASR performance, even with limited dysarthric speech data, making it a robust solution for DSR challenges. Traditional DSR systems generally employ conventional noise reduction techniques that are not specifically optimized for the distortions present in dysarthric speech, often failing to adequately enhance speech clarity. In contrast, the proposed model integrates the S-SEGAN as a front-end processing stage, significantly enhancing speech clarity by combining SEGAN’s adversarial learning strengths with SepFormer’s advanced speech separation capabilities, effectively reducing noise and distortions.

The following describes the major contributions of the proposed work:

i. *S-SEGAN*: DSE is performed by SepFormer-SEGAN. It reconstructs a cleaner speech signal from the distorted dysarthric input using the combined capabilities of SepFormer and the SEGAN. This improved signal facilitates more accurate DSR.
ii. *T-HAN*: This model combines the Transformer’s ability to capture long-range dependencies in speech sequences with the multilayered attention mechanism of an HAN. This allows T-HAN to effectively prioritize both the overall context and finer details within dysarthric speech.
iii. *C-HAN*: Conformers excel at modeling short-term and long-term speech dependencies. By combining this with HAN’s multilayered attention, C-HAN effectively captures the nuanced and disrupted patterns of dysarthric speech. Additionally, C-HAN is computationally efficient, making it suitable for limited dysarthric speech data and eliminating the need for costly, time-consuming data augmentation techniques.

The upcoming sections detail the multistage transfer learning approach for DSR and outline the training process using the UASpeech corpus. Section "Related works" reviews related works in DSE and DSR. Section "Methodology" introduces the methodology and proposed S-SEGAN DSE architecture. Section "Proposed DSR models: T-HAN and C-HAN" discusses the architectures of the DSR models, specifically T-HAN and C-HAN. Section "Experimental setup" outlines the experimental setup. Finally, Section "Results and discussion" compares the current study with previous research and evaluates the performance of the DSE and DSR models.

## Related works

Speech enhancement (SE) techniques can be broadly divided into two main categories. Traditionally, the classical SE approach involves enhancing speech time–frequency (TF) representations, such as spectrograms, which include most model-based and recent deep learning techniques^35,36^. Recently, new methods have been developed to enhance raw speech time-domain waveforms directly, bypassing transformational processes. GANs have been used in speech enhancement tasks both without attention mechanisms and with attention mechanisms^37,38^. It has also been employed to improve the robustness of ASR models. Traditionally, speech enhancement and recognition systems are created separately, with the enhancement system optimized using metrics that do not necessarily align with the final performance of the ASR system^39–41^. In recent research, the significance of phase in enhancing the quality of denoised speech has been emphasized^42,43^. For example, the SEGAN was developed as an adversarial framework to convert noisy speech waveforms into denoised speech. Various SEGAN adaptations have been proposed to improve the generator’s performance and integrate an additional TF domain loss for combined domain advantages. In the realm of DSR, GANs have shown promising potential to address the unique challenges posed by dysarthric speech patterns. Dysarthric speech often exhibits variability and distortions that traditional ASR systems struggle to handle. By leveraging GANs, researchers have been able to enhance dysarthric speech quality, making it more intelligible for recognition systems^44,45^. Dysarthria voice conversion systems often encounter challenges that require a lot of data from both the person with dysarthria and the desired speaker. Collecting this much data can be difficult and impractical for patients. To overcome this, a method called DVC 3.1 has been developed. It uses a combination of text-to-speech technology and Star GAN-VC architecture^46,47^. Despite ASR being a cornerstone in dysarthric Speech Communication Rehabilitation (SCR), recent research highlights an urgent need for advancements in dysarthric speech recognition^48–50^. Numerous methodologies employ transformers to capture long-term dependencies within both the waveform and the spectrogram^51^. Dong et al.^52^ were the first to introduce Transformer and attention-based models to ASR, implementing a 2-D attention mechanism and testing it on the Wall Street Journal’s standard speech corpus. An interpolation-based approach is investigated to generate a prior acoustic model from a model trained on standard speech. This resulting model can be adapted and employed with efficacy in the realm of dysarthric speech^53^. Cognition-inspired feature Decomposition and Recombination Network (CFDRN) leverages insights from human auditory processing to improve DSR. Recent developments have seen conformers emerge as an alternative to transformers in both ASR and speech separation tasks. Their capacity to capture both local and global contexts makes them particularly appealing^54^. End-to-end models can directly learn the relationship between dysarthric acoustic features and their corresponding words, bypassing the need for linguistic features. This can lead to higher recognition accuracy, especially for severe dysarthria cases where traditional feature-based methods struggle^55^. The Dysarthric Speech Transformer utilizes a specialized deep transformer architecture. To address the issue of limited data, it implements a two-phase transfer learning process that makes use of healthy speech. This process also investigates various neural freezing configurations and includes audio data augmentation techniques^32^. Consequently, conformers have also been applied in both time-domain and TF-domain SE tasks^56^. Conformers can be adapted for various speech recognition tasks, including speaker identification, diarization and end-to-end ASR^57–59^. In^60^, the authors analyzed the impact of dilated convolutions, a specific type of convolutional layer, within conformers for speech recognition. The literature indicates that traditional DSR systems often use generic noise reduction techniques that fail to address the unique distortions in dysarthric speech. The proposed model improves speech clarity by integrating S-SEGAN for noise reduction and SepFormer for speech separation, significantly enhancing recognition accuracy. Unlike traditional systems that rely on limited dysarthric speech datasets, this approach uses a multistage transfer learning strategy. Initially, T-HAN and C-HAN models are trained on a large dataset (LibriSpeech) and then fine-tuned on dysarthric speech, that simulates dysarthric characteristics, helping bridge the gap between typical and dysarthric speech. This method enhances model robustness and performance by capturing long-range dependencies and improving spectral feature interpretation. This architecture, selected for its efficiency, boosts word recognition accuracy and proves more practical than training from scratch, especially given the limited availability of dysarthric data.

## Methodology

**Fig. 1:**
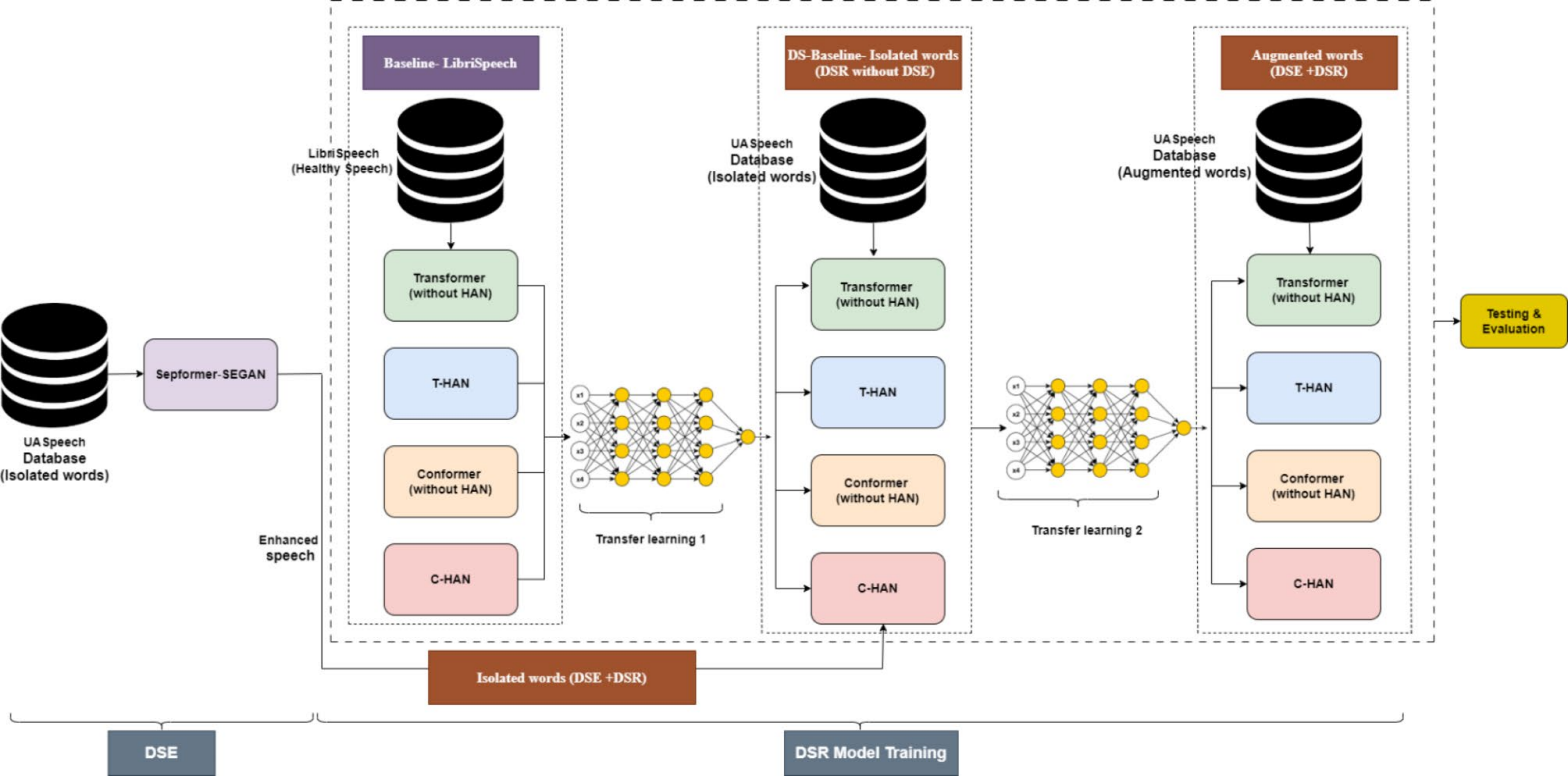
Proposed methodology overview.

Figure 1 illustrates the comprehensive methodology and training pipeline. SepFormer has demonstrated exceptional ability in capturing long-range dependencies within audio sequences^61^. By integrating SepFormer into the SEGAN framework, SepFormer-SEGAN aims to achieve significantly more accurate reconstructions of clean speech from dysarthric recordings. This enhanced speech will then be fed into powerful recognition models for DSR accuracy. Although the Transformer and Conformer models have achieved impressive results in standard speech recognition tasks, they may not be adequate on their own for recognizing dysarthric speech^62^. To address these limitations, we propose integrating a HAN within the Transformer and Conformer architectures. HAN’s layered attention addresses both individual words (local) and their sentence relationships (global). This is crucial for dysarthric speech, where subtle cues matter. By considering both local and global features, HAN creates a more nuanced speech representation, even with dysarthria, leading to improved recognition accuracy.

### Proposed S-SEGAN for DSE

S-SEGAN is specifically tailored for dysarthric speech by integrating speech enhancement techniques with advanced separation frameworks to handle the unique challenges posed by dysarthric audio signals. Dysarthric speech is often characterized by irregular articulation, breathiness, and varying speech intelligibility, which complicates the task of cleanly separating and enhancing these signals in noisy or multi-speaker environments. To address these challenges, the generator ‘G’ in SEGAN is adapted to focus on enhancing dysarthric speech by learning to denoise and improve the overall clarity of the input audio. This improvement is especially crucial for dysarthric speech, which frequently includes distortions and irregular acoustic patterns that conventional ASR models find challenging to process. The generator’s architecture is designed to capture the subtle features in dysarthric speech, such as variations in intensity and frequency content, helping improve the quality before the separation process begins. Once the speech is enhanced, SepFormer, with its masking-based separation network, processes the enhanced signal. SepFormer’s encoder divides the enhanced audio into distinct representations, and its masking network isolates individual speaker components. For dysarthric speech, the integration with SepFormer allows the system to handle complex and overlapping speech patterns, which are common in dysarthric individuals, especially in multi-speaker scenarios. SepFormer’s proficiency in source separation ensures that despite the irregularities in dysarthric speech, the model can accurately separate multiple speakers or focus on the target speaker’s voice. The discriminator ‘D’ in SEGAN evaluates the quality of the enhanced speech during training, ensuring that the generated signals maintain intelligibility and sound natural. For dysarthric speech, the discriminator is trained to distinguish between clean and distorted dysarthric signals, further refining the generator’s ability to enhance speech in a way that preserves critical speech characteristics.

#### Network architecture of speech enhancement GANs (SEGANs)

**Fig. 2:**
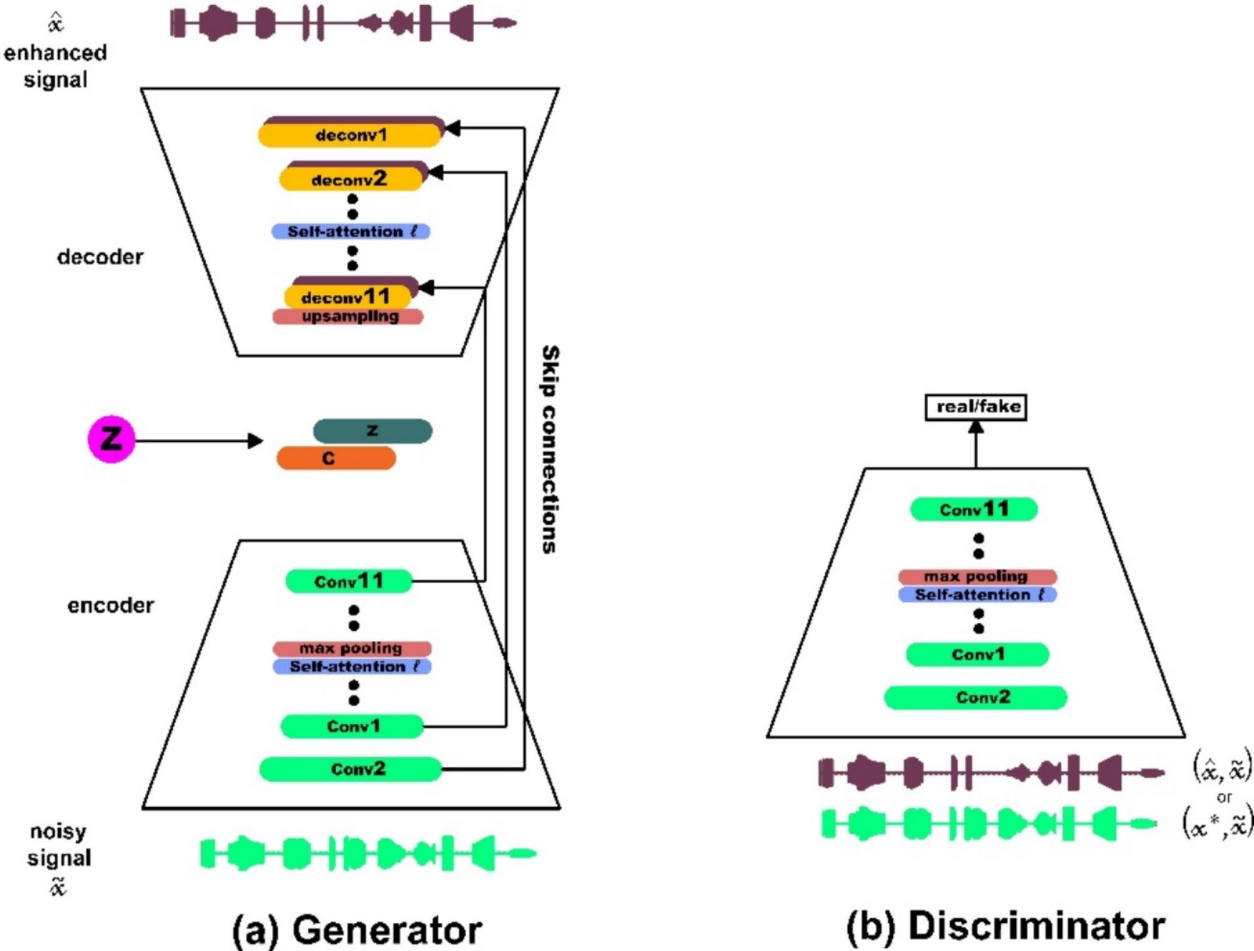
Architecture of the SEGAN: (**a**) Generator and (**b**) Discriminator.

The SEGAN, introduced by Pascual et al.^30^, consists of two primary components: a generator and discriminator. Generator is specifically designed to be fully convolutional, aiming to minimize training parameters and reduce training time by excluding compact layers^48^. The preprocessed signal ‘A’, with dimensions of 128 × 128, is initially fed into a Fully Connected (FC) layer, which transforms it into a 256 × 64 output to stabilize the training process. This output is then reshaped and processed through rectified linear units (ReLUs), followed by another reshaping into a 4 × 4 × 1024. Subsequently, the signal passes through five ReLU layers, each generating a 128 × 128 output, and is finally processed by a transposed convolution layer. The generator ends with a tanh activation layer, which produces the output signal. The discriminator then takes this 128 × 128 × 1 output as input. It includes five convolutional layers followed by leaky ReLU activations and reshapes the output to 64 × 256. The final FC layer computes the signal 'R' with dimensions of 1 × 1. Figure 2a and b depict the architectures of the generator and discriminator, respectively, illustrating the SEGAN structure.

Given a dataset $X=\left\{\left({x}_{1}^{*}, {x}_{1}^{\sim }\right),\left({x}_{2}^{*}, {x}_{2}^{\sim }\right),\dots \left({x}_{N}^{*}, {x}_{N}^{\sim }\right)\right\}$ consisting of $N$ pairs of signals, where ${x}^{*}$ represents clean speech signals and $\widetilde{x}$ represents noisy speech signals, the objective of speech enhancement is to find a mapping ${f}_{\theta }\left(\widetilde{x}\right):\widetilde{x} \to \widehat{x}$ that transforms a noisy signal $\widetilde{x}$ into an enhanced signal $\widehat{x}$. Here, θ denotes the parameters of the enhancement network. Following GAN principles, the generator aims to learn a mapping that mimics the real data distribution, generating new samples that resemble those in the training set. Thus, generator functions as the enhancement module. Moreover, the discriminator, acts as a classifier, distinguishing between real samples from the actual dataset and fake samples produced by generator. The discriminator guides the parameters (θ) toward the distribution of clean speech signals. Specifically, within the SEGAN framework, generator executes the enhancement mapping, i.e., $\widehat{x}=G(\widetilde{x})$, while discriminator aids in training generator by classifying pairs $({x}^{*},\widetilde{x})$ as real and pairs ($\widehat{x}, \widetilde{x}$) as fake. The ultimate goal is for generator to generate enhanced signals $({x}^{*},\widetilde{x})$ that are sufficiently realistic to deceive discriminator, resulting in D classifying ($\widehat{x}, \widetilde{x}$) as real.

#### Network architecture of the S-SEGAN

**Fig. 3:**
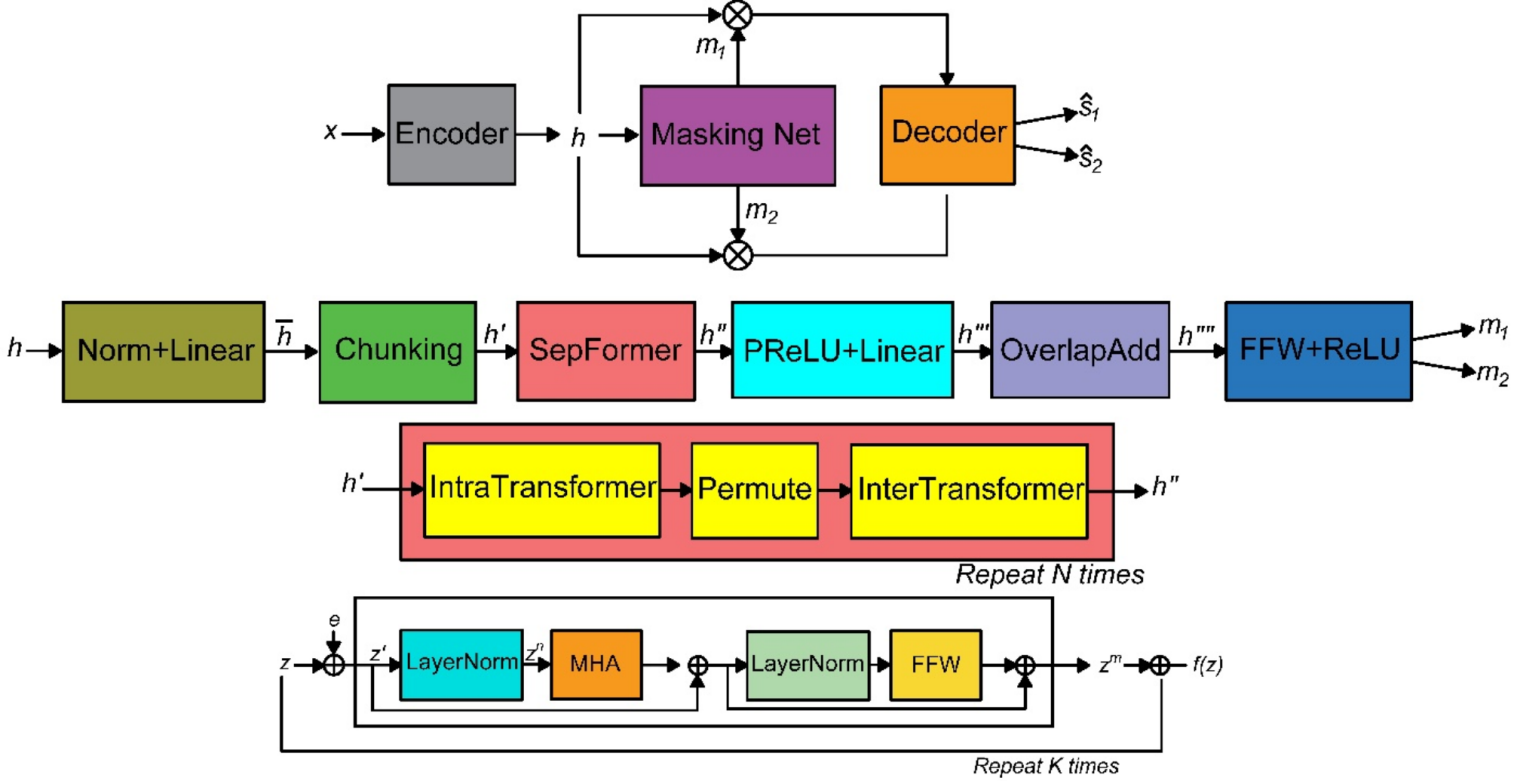
Architecture of SepFormer.

The S-SEGAN model merges SepFormer and SEGAN for enhanced speech processing. SepFormer employs a transformer architecture to effectively separate speech from noise, while SEGAN uses adversarial training with a generator and discriminator to further refine the enhanced speech. This combination leverages SepFormer’s advanced separation capabilities and SEGAN’s adversarial learning to produce high-quality speech. SepFormer employs a masking-based source separation framework, as shown in Fig. 3, which has been widely adopted in recent research^63^. The encoding process begins with the input of the time-domain mixture signal $\left(x \in {R}^{T}\right),$ where *x* represents the input audio signal that contains a mixture of sounds from multiple speakers. The parameter ‘*T*’ denotes the length of the time-domain signal, which is the number of time samples in the audio input. Through a single convolutional layer, the encoder learns to generate a representation $\left(h \in {R}^{F \times T^{\prime}}\right),$ akin to a short-time Fourier transform (STFT). The equation for this process is

1 $$h =\text{ ReLU}\left(conv1d-\left(x\right)\right)$$

where ‘ℎ’ is the encoded representation in the time–frequency domain. *F* represents the number of frequency bins or channels after the convolution. *T***′** is the transformed time dimension, which depends on the stride and kernel size of the convolutional layer. The stride parameter in this convolution significantly influences the model’s performance, speed, and memory utilization. The masking network then processes the encoded representations $\left(h \in {R}^{F \times T^{\prime}}\right),$ to compute masks $\{m1 ,\dots ,mNs \}$ for each of the *N*_*s*_ speakers in the mixture. The encoded input ‘ℎ’ undergoes layer normalization and linear transformation, followed by chunking into overlapping segments of size ‘C’. This chunking results in a representation $\left({h}^{\prime} \in {R}^{F \times C\times {N}_{C}}\right),$ where ‘C’ is the length of each chunk. N_*c*_ is the number of chunks formed. These chunks are fed into the SepFormer block, which employs two types of transformers, an intra-transformer (IntraT) and an inter-transformer (InterT), to learn short-term and long-term dependencies, respectively. The output from SepFormer is denoted as ${h}^{{\prime}{\prime}} \in {R}^{F \times C\times {N}_{C}}$. PReLU activations and linear layers further process this output to form $\left({h}^{{\prime}{\prime}{\prime}} \in {R}^{(F \times {N}_{s})\times C\times {N}_{C}}\right),$ and, through an overlap-add scheme, to yield $\left({h}^{{\prime}{\prime}{\prime}} \in {R}^{F \times {N}_{s}\times {T}^{\prime}}\right)$. The final masks *m*_*k*_ for each speaker are produced by passing ${h}^{{\prime}{\prime}{\prime}}$ through additional feed-forward layers and ReLU activation. The transformation can be expressed as

2 $$h^{\prime } = f_{inter} (f_{intra} (h^{\prime } )))$$

where ${f}_{inter}$ denotes the InterTransformer block and ${f}_{intra}$ denotes the IntraTransformer block. P represents the permutation operation for interchunk processing. This SepFormer transformation is repeated *N* times within the block. For the decoding process, a transposed convolution layer is used, which mirrors the encoder’s parameters. The decoder’s input is the elementwise multiplication of the source mask and the encoder output $h$, described by

3 $$\widehat{{s}_{k}}=conv1d-transpose({m}_{k}*h)$$

where $\widehat{{s}_{k}}\in {R}^{T}$ represents the source separation $k$.

## Proposed DSR models: T-HAN and C-HAN

### Network architecture of T-HAN

**Fig. 4:**
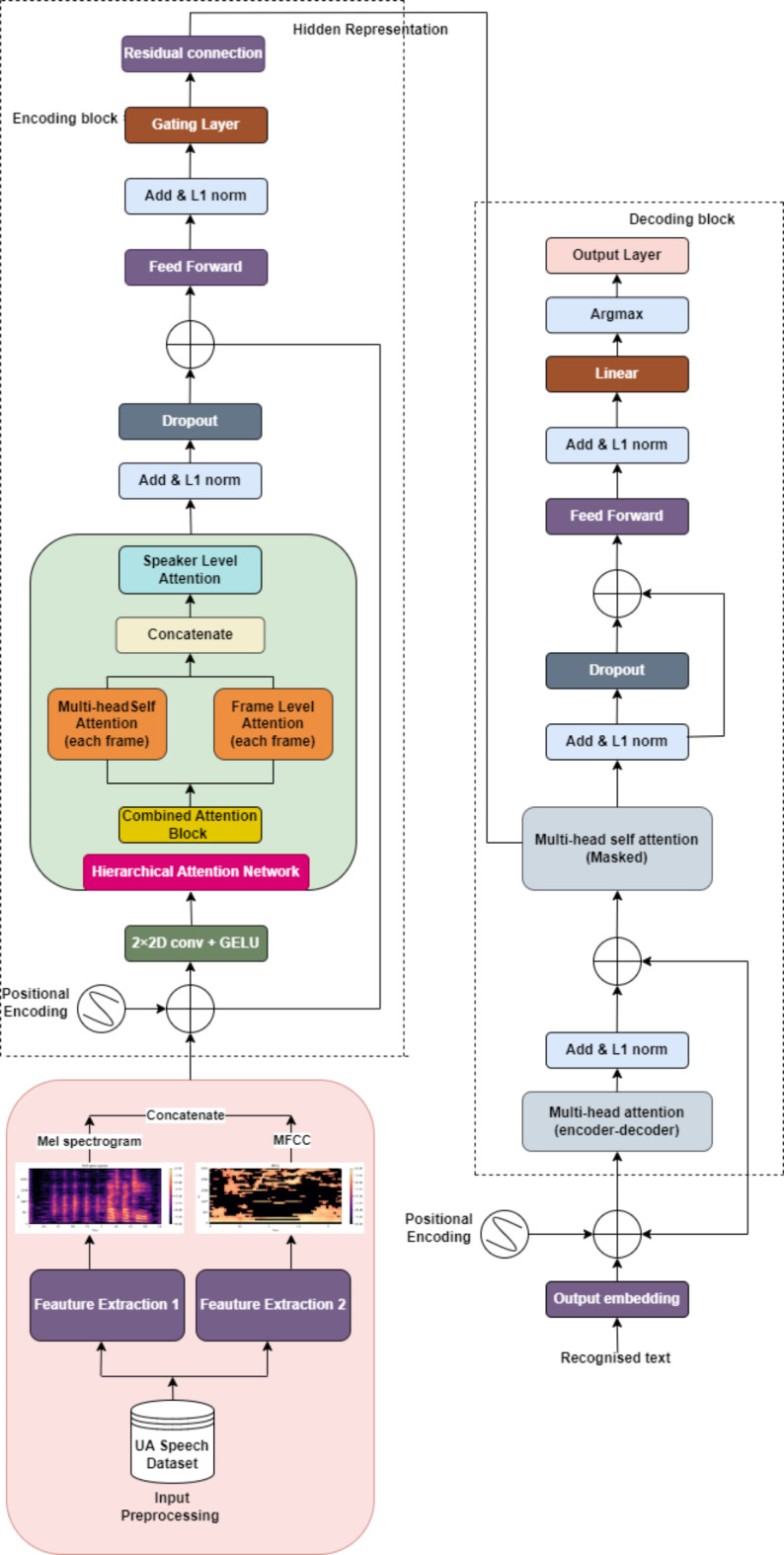
Architecture of the transformer with the hierarchical attention network (T-HAN).

The architecture of the T-HAN is composed of four encoder modules and one decoder module, as shown in Fig. 4. In preparation for the encoder section, input is obtained through Mel-Frequency Cepstral Coefficients (MFCC) and Mel spectrograms tailored specifically for dysarthric speech. The MFCC and Mel spectrograms are then passed through a 2D convolutional layer with 64 filters of 3 × 3 kernel size and a stride of 1. This configuration allows the convolutional layer to extract spatial and temporal features simultaneously, capturing both short-term and long-term patterns inherent in dysarthric speech. The utilization of the Gaussian error linear unit (GELU) activation function offers a significant advantage over traditional activation functions. Its smooth, nonlinear nature allows the model to better capture the complex and potentially distorted features present in dysarthric speech. Compared with the 1D convolutional layers, the 2D convolutional layer preserves the spatial relationships between different frequency bands and time frames, enabling the model to discern intricate spectral and temporal variations present in dysarthric speech. The speech features from the convolution layer are provided to the multihead attention layer for processing. In this multihead attention layer setup, we utilize three heads to process the input speech features. By leveraging multiple heads, the network can effectively learn and represent complex speech patterns. The input data, represented as a Mel spectrogram, are projected into query, key, and value vectors. This transformation is typically realized through linear transformations implemented as fully connected layers, where learnable weight matrices are applied. Specifically, the linear projections yield query $Q$, key K and value $V$ matrices. Mathematically, these projections can be expressed as follows: $Q= X{Q}_{w}K= X{W}_{k},$ $V= X{W}_{v}$ and where $X$ represents the input data and ${Q}_{w}, {W}_{k}, and {W}_{v}$ denotes the learnable weight matrices corresponding to the query, key, and value transformations, respectively. For each attention head $i$, the attention scores ${A}_{i}$ between Q and K are computed as

4 $$A_{i} = Softmax \left( {{\raise0.7ex\hbox{${QW_{Qi} (KW_{Ki} )^{T} }$} \!\mathord{\left/ {\vphantom {{QW_{Qi} (KW_{Ki} )^{T} } {\sqrt {d_{k} } }}}\right.\kern-0pt} \!\lower0.7ex\hbox{${\sqrt {d_{k} } }$}}} \right)$$

Here, ${W}_{Qi}$ and ${W}_{Ki}$ are learnable weight matrices specific to the attention head $i$, and ${d}_{k}$ is the dimensionality of the key vectors. The outputs of the individual attention heads are concatenated, and another linear transformation is applied to derive the final multihead attention output as

5 $$MultiHead \left(Q, K, V\right)=Concat({head}_{1}, {head}_{2}, \dots ,{head}_{h}) {W}_{o}$$

Here, ${head}_{i}$ represents the output of the *ith* attention head, and ${W}_{o}$ is the output projection weight matrix. The model employs a multihead layer with a gating mechanism to enhance the focus on relevant information and suppress noise dynamically. L1 normalization ensures balanced attention weights, while masking prevents attention to padded regions. Multihead attention captures long-range dependencies crucial for DSR, while frame-level attention handles finer details within short segments. Speaker-specific characteristics are adapted using another attention mechanism, and addition followed by L1 normalization merges information sources. Dropout adds randomness for resilience to noise, and the feedforward layer captures higher-level representations. The gating layer controls the information flow, mitigating the vanishing gradient problem. In the decoder, masked attention ensures word-by-word output generation based on previously generated words and encoded speech features. Multi-head attention aligns decoder outputs with input sequence parts, incorporating contextual information, followed by residual connections, layer normalization, and dropout for stabilization and prevention of overfitting. The final linear layer transforms features into logits for word probabilities, iteratively generating the recognized speech sequence. Figure 4 shows the architecture of T-HAN.

### Network architecture of the C‒HAN

**Fig. 5:**
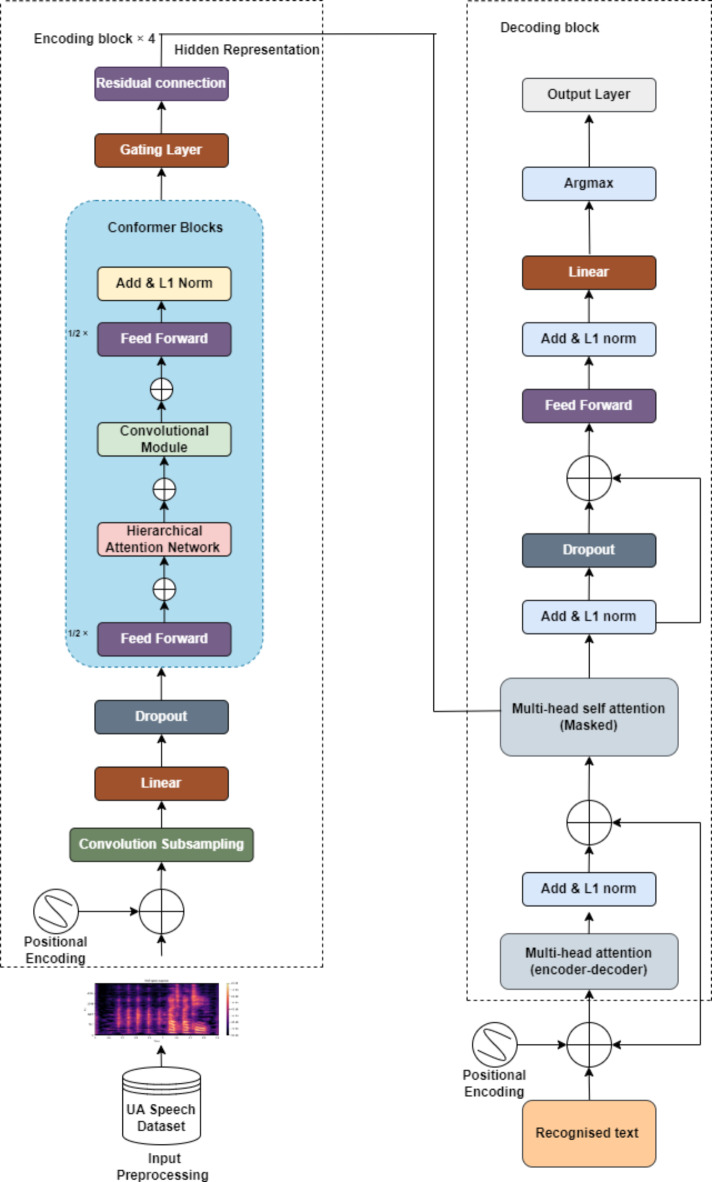
Architecture of the Conformer with the Hierarchical Attention Network (C-HAN).

**Fig. 6:**
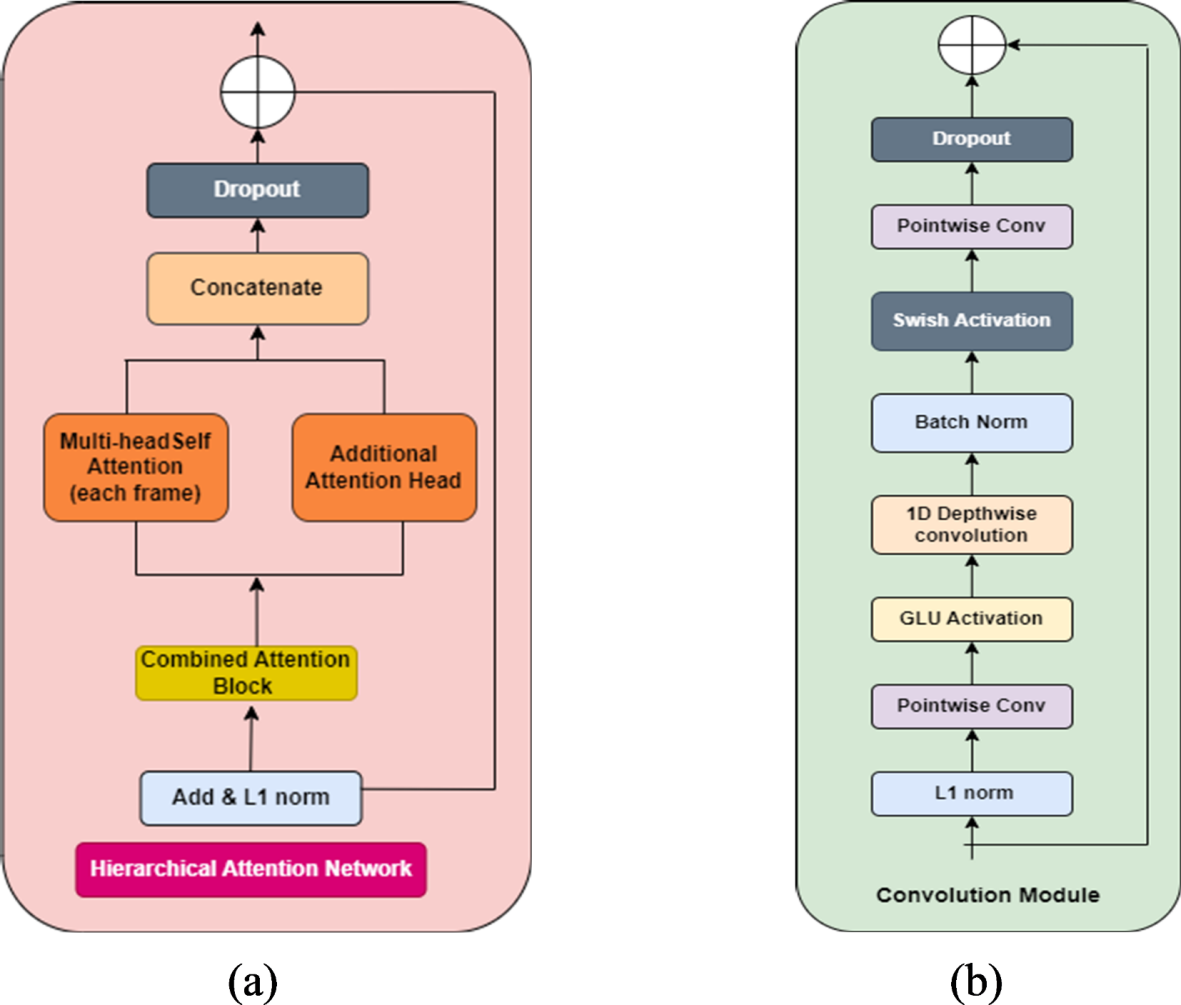
(**a**) Hierarchical attention network of the conformer, (**b**) Convolutional module of the conformer.

The Conformer is a state-of-the-art ASR encoder architecture that diverges from the standard Transformer block by incorporating a convolution layer to enhance local information modeling within the Transformer encoder model^49^. The architecture of the C-HAN is composed of four Conformers and one Transformer decoder, as shown in Fig. 5. The encoder processes input through a convolutional subsampling layer followed by Conformer blocks. (i) Each Conformer block integrates a HAN module, as depicted in Fig. 6a; a convolution module, as shown in Fig. 6b; and two magnetic-feedforward (FFN) modules. The HAN module, comprising an additional attention block and MultHead Self-Attention (MHSA) for each frame, is designed to capture contextual information at multiple levels. This hierarchical attention mechanism improves the model’s ability to handle varying temporal dependencies and enhances overall accuracy by applying different layers of attention. Each layer focuses on different granularities of the input sequence, thus providing a more comprehensive understanding of the input data. Each module in the Conformer block is preceded by layer normalization, followed by dropout and residual connections. The convolution module begins with a 1D pointwise convolution layer, followed by Gated Linear Unit (GLU) activation. This convolution layer increases the number of input channels, while GLU divides the input across channels, enabling more intricate feature interactions through elementwise multiplication. Then, a 1D depth wise convolution layer, batch normalization layer, Swish activation layer, and another 1D pointwise convolution layer sequentially follow. Swish activation is chosen for its smooth, non-monotonic properties, enhancing gradient flow and training stability compared to ReLU. GLU is employed for its ability to introduce gating mechanisms that regulate information flow, improving the model’s ability to capture complex patterns. Unlike the FFN module in the Transformer encoder, which includes two linear transformations separated by ReLU activation, the Conformer encoder incorporates an additional FFN module and replaces ReLU with Swish activation. Inspired by Macaron-Net^64^, these FFN modules function in a half-step manner, surrounding the MHSA and convolution modules. The output ${o}_{i}$ of the Conformer is expressed as

6 $${m}_{i}^{\prime}={m}_{i}+\frac{1}{2}FFN({m}_{i})$$

7 $${m}_{i}^{{\prime}{\prime}}={m^{\prime}}_{i}-MHSA({m}_{i}^{\prime})$$

8 $${m}_{i}^{{\prime}{\prime}{\prime}}={m^{{\prime}{\prime}}}_{i}+Conv({m}_{i}^{{\prime}{\prime}})$$

9 $${o}_{i}=Layer norm \left({{ m}_{i}^{{{\prime}{\prime}}{\prime}}}+ \frac{1}{2}FFN \left({{m}^{{\prime}{\prime}{\prime}}}_{i}\right)\right)$$

where FFN (·), MHSA (·), Conv (·), and Layer Norm (·) represent the macron-feedforward module, the multihead self-attention module, the convolution module, and the layer normalization module, respectively.

## Experimental setup

### Database

The availability of publicly accessible speech corpora containing dysarthric speech samples is noticeably limited. UA-Speech^65^ is the largest dataset with a significant number of dysarthric participants. It has been widely utilized in DSR research and is publicly available. Therefore, the UA-Speech dataset was chosen as the primary dataset for this study. Developed by researchers at the University of Illinois, UA-Speech is a comprehensive dataset featuring recordings from 19 individuals with dysarthria exhibiting varying levels of speech intelligibility ranging from 2 to 95%. These levels are categorized from very low (0–25%) to low (25–50%), mild (50–75%), and high (75–100%) intelligibility. In a controlled laboratory environment, each dysarthric participant uttered 765 individual words. The UA Speech dataset consists of blocks B1 and B2, which are designed as subsets that are specifically tailored for training speech recognition models. B1 focuses on common words (CMs), such as ten digits (zero to nine), computer commands (e.g., enter, delete), and 26 radio alphabets (e.g., bravo, alpha), providing foundational training. Conversely, B2 incorporates uncommon words (UW) (e.g., unusual, atrocious, exploit) to refine model performance. While B1 emphasizes frequently spoken terms, B2 expands the model’s vocabulary to include fewer common terms, ensuring adaptability to diverse speech contexts. This dataset presents a distinctive compilation comprising 300 uncommon words, three iterations of 26 letters from the radio alphabet, 19 computer-related commands, 10 numerical digits, and 100 commonly used words, offering a diverse range of linguistic content. Data collection involved audio recordings conducted at a sampling rate of 16 kHz, employing an array of 8 microphones, with one dedicated to synchronization purposes. As a result, each word was recorded a total of 21 times (3 repetitions multiplied by 7-word categories). For training and evaluating the Transformer and Conformer models for word-level and sentence-level DSR, a multistage transfer learning approach is employed. In first stage, the models are trained on the diverse and high-quality LibriSpeech train-clean dataset (Baseline), providing diverse data from approximately 960 speakers with high-quality recordings. This training helps the models capture fundamental patterns and features of spoken language. In second stage, they are fine-tuned on the UA-Speech dataset, adapting to the specific challenges of dysarthric speech. This involves adapting the pre-trained models to the specific characteristics of dysarthric speech, which includes handling variability in phoneme production, distortion, and background noise. This two-phase process blends broad speech understanding with targeted adaptation, optimizing performance in recognizing dysarthric speech. The dataset utilized in the proposed model employs a 75:25 train/test split for both healthy speech (LibriSpeech) and UA-Speech. Recognizing the complexity of fitting the extensive UA Speech database, which consists of 66,280 speech signals, into a model, we have taken steps to streamline accessibility. The dataset has been meticulously preprocessed and privately uploaded to the Hugging Face platform with train and test splits. We have also implemented a streamlined procedure for converting the audio folder format to Hugging Face. Additionally, it’s worth noting that access to the dataset will be granted based on a request to ensure responsible usage and adherence to ethical guidelines. Given the scarcity of publicly available sentence-level dysarthric speech data and the lack of corresponding transcripts, we augmented the UASpeech dataset for training and testing at the sentence level, as shown in Table 1. Here, we breakdown how the model utilizes the dataset:

- Word-level DSR: We began by fine-tuning the baseline model on isolated common words from the UASpeech dataset.
- Sentence-level DSR: Next, we further fine-tuned the model using the UASpeech dataset augmented with common words (as detailed in Table 1).
- Generalization Assessment: Finally, we evaluated the model’s ability to generalize by testing it on the UASpeech dataset under two conditions: with isolated uncommon words and with the dataset augmented with both common and uncommon words, as listed in Table 1.

**Table 1:** Few examples of augmented common and uncommon words used in model training and testing.

Augmented sentence (common words)	Augmented sentence (uncommon words)
One cup of tea One yellow banana Enter the command Delete the paragraph He has no time Where are you from Look at you She is a gentlewoman Tree is immovable Write the command She giggled Can I go now Monkey is gigantic I will delete that sentence How long you have been in call I did my autobiography with Joseph Bravo have my yellow cup with him They cut the banana with knife She is from Asia Have a good day I have one banana	Swimming is joyful I found butterflies in schoolyard I have toothache Ha-ha Charlie giggled I have one green crayon Bravo, you did a good job She is crying He is thirty-five Joseph is a good entertainer Judith overshadowed Joseph I have treehouse Victor is lawyer He is bachelor She is atrocious I have ten watches Bungalows are made up of glasses Orange is good He got an output in python She is ashamed of my behavior He shout at me I have eight balloons

### Training of the dysarthric speech enhancement model: S-SEGAN

**Fig. 7:**
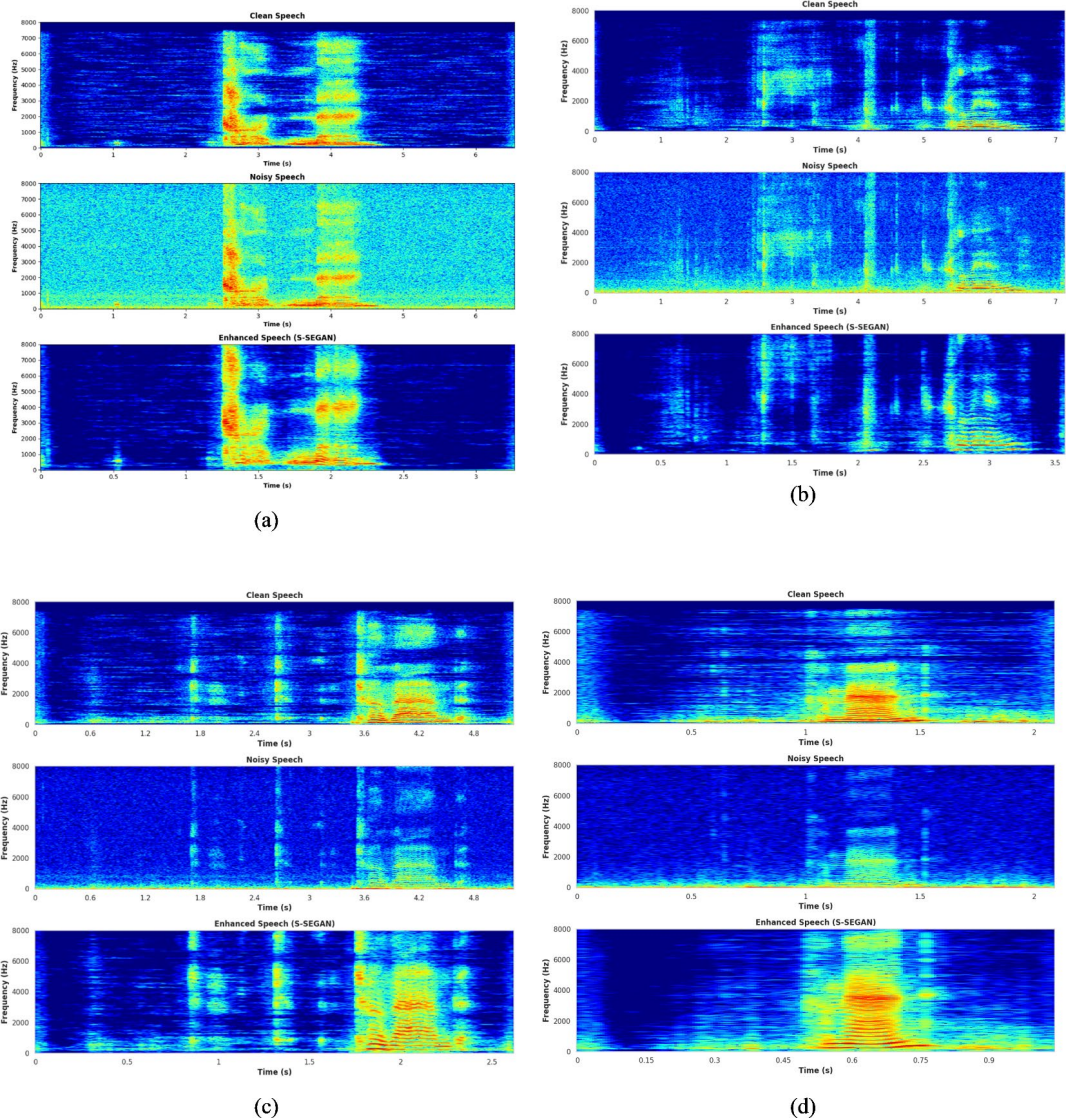
(**a**) Mel spectrogram of clean, noisy and S-SEGAN-enhanced speech: Speaker F02 (Low); (**b**) Mel spectrogram of clean, noisy and S-SEGAN-enhanced speech: Speaker M01 (Very low); (**c**) Mel spectrogram of clean, noisy and S-SEGAN-enhanced speech: Speaker M05 (Mild); (**d**) Mel spectrogram of clean, noisy and S-SEGAN-enhanced speech: Speaker M08 (High).

All experiments were performed on our custom deep learning workstation, a Lenovo Think Station equipped with an Intel Xeon processor, 128 GB of RAM, and four NVIDIA Quadra RTX 8000 GPUs. The input to the S-SEGAN model will be mixed speech samples, combining clean dysarthric speech with noisy dysarthric speech from the UA Speech dataset to simulate real-world environments. The original UA Speech dataset offers a distinct advantage: it contains both clean dysarthric speech and recordings with background noise (i.e., noisy dysarthric speech below 20 dB). This eliminates the need for introducing additional artificial noise during the training phase. The clean speech from the UA Speech dataset is used as the target output for the model. Figure 7(a–d) shows spectrograms representing clean speech, noisy speech, and S-SEGAN-enhanced speech. The generator includes three convolutional layers with channel numbers set to 4, 4, and 64. The discriminator, known as the metric discriminator, comprises convolutional layers with channel numbers set to {16, 32, 64, and 128}. The S-SEGAN model is trained for 40 epochs using the Adam optimizer with a learning rate of 0.001. The batch size is set to 32. During training and testing, we apply a high-frequency pre-emphasis filter (coefficient: 0.97) to all the input signals. Within the SepFormer component, self-attention mechanisms integrated into its Transformer-based architecture are utilized to improve the discrimination between speech components and noise. These mechanisms optimize performance while ensuring computational efficiency. The F-Bank feature extraction network transforms enhanced speech from S-SEGAN to DSR. We extracted 64-dimensional filter-banks (20 ms window, 10 ms shift), adding temporal first- and second-order differences. Next, logarithmic calculation is conducted followed by global mean–variance normalization as defined in Eq. (10).

10 $$\widehat{f}=FBank\left(\widehat{x}\right)=Norm(\text{log}(Mel\left(STFT\left(\widehat{x}\right)\right)))$$

The F-Bank feature extraction layer starts with the short-time Fourier transform (STFT), which converts the raw waveform into its frequency domain representation. Following this, the Mel matrix multiplication operation, denoted as Mel (·), is applied to the STFT outputs, which involves mapping the STFT coefficients onto the Mel frequency scale. Finally, the output undergoes normalization using the norm (·) operation, which separately normalizes the mean and variance to 0 and 1, respectively. In the proposed T-HAN DSR model, we utilize both the Mel spectrogram and MFCC for feature extraction. To extract MFCC features, we extend the process outlined for the Mel spectrogram, as given in Eq. (11). Where $\widehat{x}$ is the enhanced speech signal from the S-SEGAN model. $DCT$ computes the discrete cosine transform to obtain the MFCC.

11 $$\widehat{f}=MFCC\left(\widehat{x}\right)=DCT(\text{log}\left(Mel\left(STFT\left(\widehat{x}\right)\right)\right))$$

### Training of dysarthric speech recognition models: T-HAN & C-HAN

(i) T-HAN

**Fig. 8:**
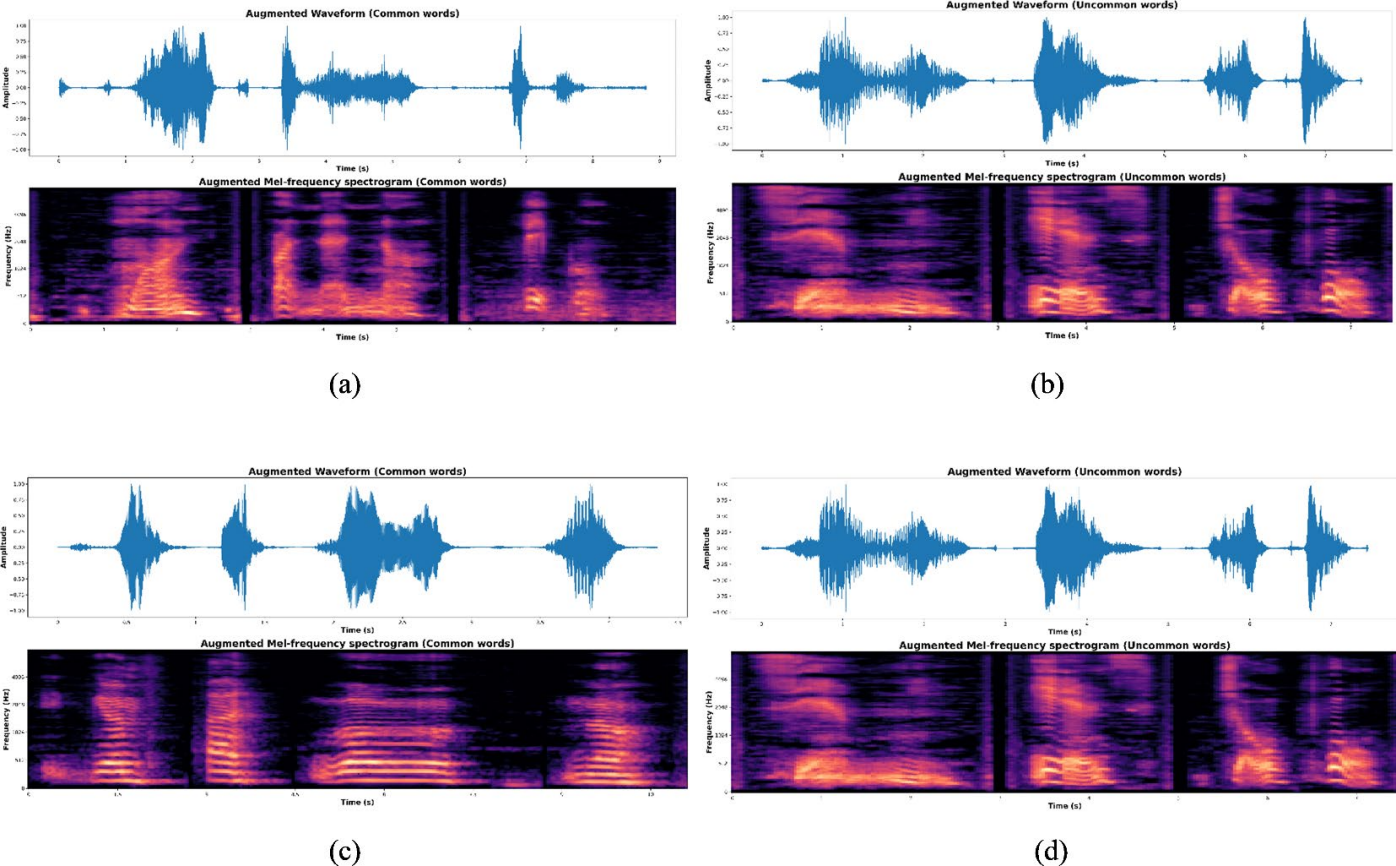
(**a**) Augmented common words of S-SEGAN-enhanced speech: Speaker F02 (Low); (**b**) Augmented Uncommon words Spectrogram of S-SEGAN-enhanced speech: Speaker M01 (Very low); (**c**) Augmented Common words of S-SEGAN-enhanced speech: Speaker M05 (Mild); (**d**) Augmented Uncommon words of S-SEGAN-enhanced speech: Speaker M08 (High).

The T-HAN model is initially trained on the clean training dataset from LibriSpeech, establishing a baseline performance. To significantly improve the model’s ability to recognize crucial features in dysarthric speech, the baseline model is fine-tuned using enhanced dysarthric speech data generated by the S-SEGAN model. These data specifically focus on isolated common words. To further refine the model’s capabilities, the model undergoes further refinement utilizing a dataset comprising 10,000 augmented sentences derived from isolated common and uncommon words. The training set included 7621 augmented utterances, and the test set contained 2379 augmented utterances. Various augmentation techniques are employed in this process. First, speech parameter perturbations are applied, including pitch shifting to subtly alter the fundamental frequency of words and time stretching to simulate varying speech speeds encountered in dysarthric speech. Additionally, silence trimming meticulously removes unnecessary leading and trailing silence from recordings to ensure accurate segmentation. Sentence construction with delays is then implemented, where augmented words are sequenced to form grammatically correct sentences (e.g., "one yellow banana"), with variable delays inserted between words to mimic natural speech patterns and pauses, as shown in Fig. 8(a-d). These techniques helped reduce overfitting and improved model robustness, enabling better generalization across speakers. For optimization, the Adam optimizer is employed, with meticulous configuration parameters such as β1 = 0.9, β2 = 0.98, and ε = ${10}^{-9}$. The learning rate follows a warm-up strategy, where it increases linearly for the first 15,000 steps. After this initial phase, it transitions to a decay schedule, decreasing in proportion to the inverse square root of the step number. To combat overfitting, we introduced residual dropout and attention dropout mechanisms, each with a rate of 0.1, targeting sub-blocks and softmax activations, respectively. Additionally, we devised a novel regularization approach designed to enhance the model’s attention to nearby positions by penalizing attention weights for distant position pairs. The training regimen concluded after 35 epochs, ensuring optimal model performance while mitigating the risk of overfitting. The HAN architecture played a pivotal role in this process, efficiently capturing both local and global dependencies within speech sequences, thereby facilitating superior performance across a range of speech recognition tasks.

(i) (ii) C-HAN

The input and fine-tuning processes for the C-HAN model are the same as those for the Transformer model. The Conformer model is configured with the following hyper parameters: ${N}_{e}$=12, ${N}_{d}$=6, H = 4, ${d}_{k}$=256, and ${d}_{ff}$=2048. The convolution subsampling layer includes a two-layer CNN with 256 channels, a stride of 2, and a kernel size of 3. The convolution module features a kernel size of 31. The generator is configured with four two-stage conformer blocks (N = 4), a batch size (B = 4), and channels (C = 64). In the metric discriminator, the number of channels is set to 16, 32, 64, and 128. Dropout is applied at a rate of 0.1 in each residual unit of the Conformer. Similar to the Transformer, the network is trained using the Adam optimizer and a transformer learning rate schedule, which includes 15,000 warm-up steps. The learning rate reaches its peak at 0.05/√d, where d represents the model dimension in the Conformer encoder. The training regimen concluded after 30 epochs, ensuring optimal model performance while mitigating the risk of overfitting.

## Results and discussion

### Evaluation metrics

The evaluation of the system’s effectiveness employed a combination of objective and subjective metrics. Speech enhancement was assessed using the Signal-to-Noise Ratio (SNR), Segmental Signal-to-Noise Ratio (SegSNR, Perceptual Evaluation of Speech Quality (PESQ), Short-Time Objective Intelligibility (STOI) and Root Mean Squared Error (RMSE). The SNR is calculated as the ratio of root mean square of the speech signal to the root mean square of the noisy signal. The SegSNR is the signal power relative to the noise power for each segment of a speech signal. The PESQ is a subjective measure used to determine the quality of a signal. It is measured on a scale of 0 to 5. A value between 0–1 is considered poor, 1–2 is considered poor, 2–3 is considered fair, 3–4 is considered good, and 4–5 is considered excellent quality. The STOI is a measure used to determine speech intelligibility. The value ranges between 0 and 1. A value closer to 1 indicates better speech intelligibility. The relevant equations are given in Eqs. (12)–(14). These metrics quantified the improvements in intelligibility and signal quality achieved by the enhancement algorithm. For dysarthric speech recognition, performance was evaluated using the WER, WRA, Phoneme Error Rate (PER), Character Error Rate (CER) and Sentence Error Rate (SER). These metrics measured the accuracy of the recognition system at both the word and sentence levels.

12 $$SNR = 20 \log_{10} \frac{RMS\; of\; Speech\; signal}{{RMS\; of \;Noise\; Level}}$$

13 $$SegSNR=10{\text{log}}_{10}\frac{Sum({e}^{2})}{Sum({y}^{2})}$$

In this equation, e represents the enhanced speech signal, while y represents the noisy speech signal. RMSE refers to the square root of the mean squared error (MSE) function, defined as

14 $$RMSE = \sqrt {\frac{1}{N}\mathop \sum \limits_{n = 1}^{N} \left[ {O_{n} - R_{n} } \right]^{2} }$$

In this context, the total number of training samples is denoted as N, the target value is represented as O, and the actual output is denoted as R.

### Evaluation of the DSE model

Table 2 presents an assessment of the S-SEGAN model across various speakers, revealing differences in their performance metrics. Notably, Speaker M05 demonstrated strong performance, characterized by clear speech amidst minimal background noise, as evidenced by an SNR of 35.92 dB and an STOI score of 0.86, indicating good speech intelligibility. Conversely, Speaker M01 exhibited more modest metrics, with an SNR of 27.94 dB, suggesting a balanced speech-to-noise ratio, albeit with some difficulty in understanding speech segments, as reflected in the STOI score of 0.77. Speaker M08 emerges as a top performer, with exceptional metrics, including an SNR of 40.42 dB and an STOI score of 0.91, highlighting clear speech and high intelligibility. Speaker M09 maintains this high standard, showing strong metrics with an SNR of 41.64 dB and an STOI of 0.95. Similarly, Speaker M14 demonstrated noteworthy performance, with an SNR of 39.56 dB and an STOI of 0.93. In contrast, Speaker M16 presented more moderate metrics, suggesting a fair balance between speech and noise but with some difficulty in understanding speech segments. Transitioning to female speakers, the F05 speaker achieved impressive results, with an SNR of 41.98 dB and an STOI of 0.96, indicating exceptional speech clarity and intelligibility. Speaker F03, on the other hand, shows more room for improvement with moderate metrics, including an SNR of 29.89 dB and an STOI score of 0.75. Speaker F04 stands out among the female speakers, demonstrating superior performance metrics, notably with an SNR of 38.56 dB and an STOI of 0.89, indicating excellent speech clarity and intelligibility. Hence, the S-SEGAN model leverages DSR, providing valuable insights into the effectiveness of speech transmission across various speakers.

**Table 2:** Dysarthric speech enhancement model test results on UASpeech.

SI. No.	Speaker ID	Intelligibility level	SNR (DB)	PESQ	SEG-SNR (DB)	STOI	RMSE
1	M01	Very low	27.94	2.34	10.78	0.77	0.031
2	M04	Very low	29.43	2.65	10.89	0.79	0.036
3	M05	Mild	35.92	3.14	13.89	0.86	0.029
4	M07	Low	33.14	3.03	11.34	0.83	0.034
5	M08	High	40.42	3.88	16.94	0.91	0.014
6	M09	High	41.64	3.92	17.86	0.95	0.015
7	M10	High	40.57	3.87	16.89	0.97	0.018
8	M11	Mild	36.97	3.34	14.76	0.85	0.021
9	M12	Very low	28.98	2.87	12.21	0.74	0.026
10	M14	High	39.56	3.96	17.32	0.93	0.013
11	M16	Low	31.46	2.56	10.18	0.74	0.035
12	F02	Low	30.67	2.78	11.89	0.76	0.025
13	F03	Very low	29.89	2.34	10.52	0.75	0.029
14	F04	Mild	38.56	3.89	13.94	0.89	0.019
15	F05	High	41.98	3.98	17.92	0.96	0.011

#### Comparative study of DSE models

**Fig. 9:**
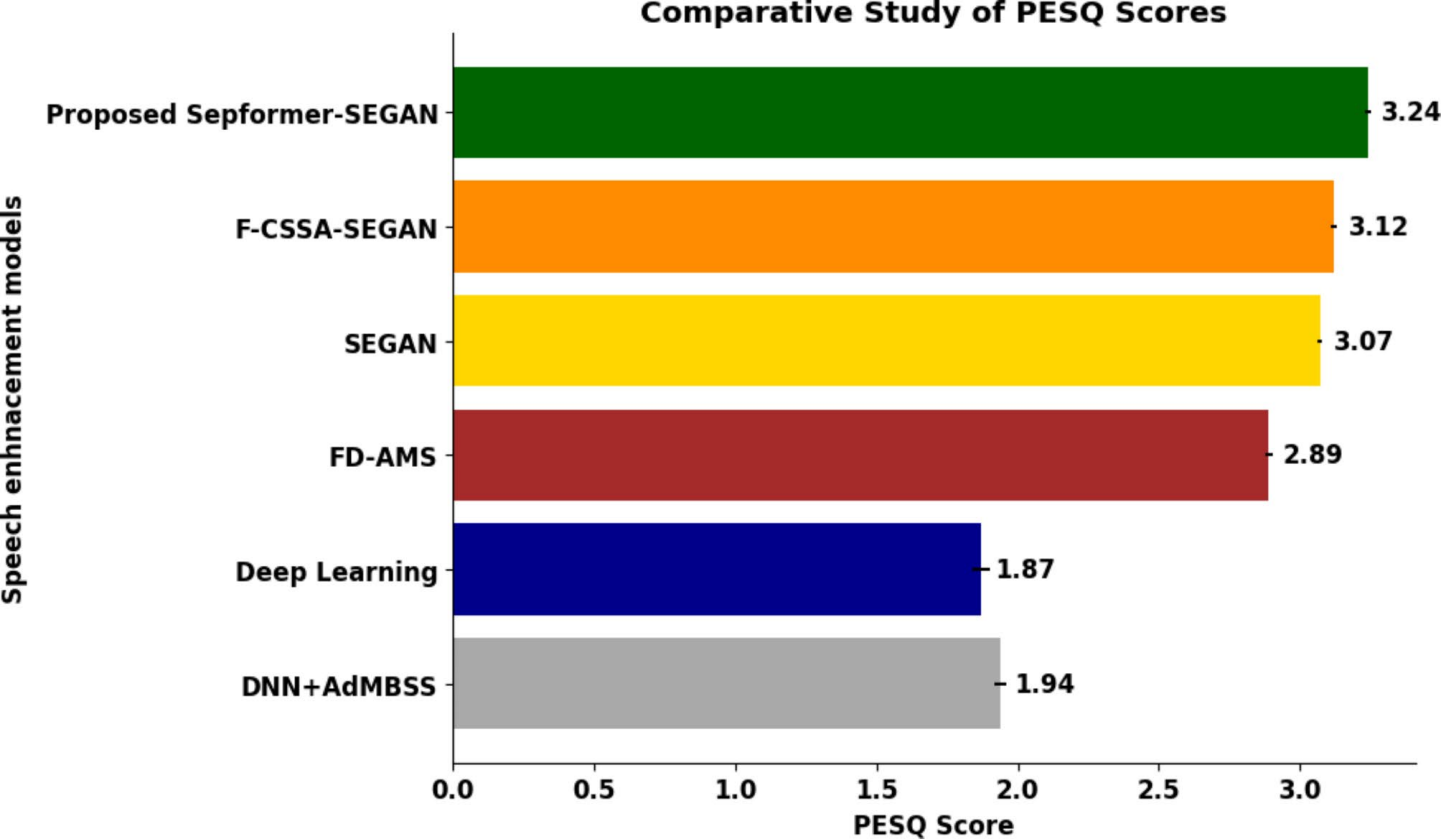
PESQ scores of the proposed S-SEGAN and existing speech enhancement models.

**Fig. 10:**
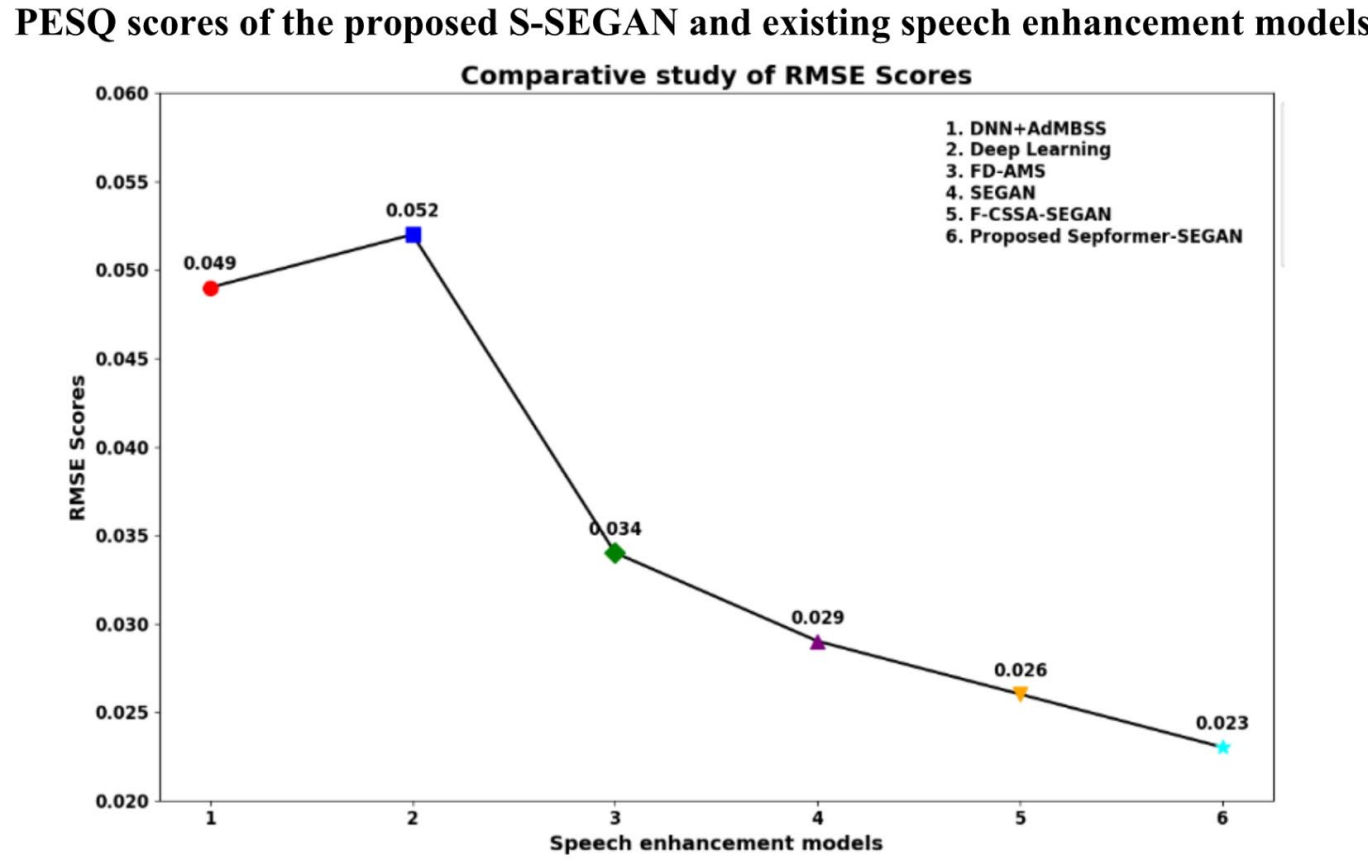
RMSE of the proposed S-SEGAN model versus the existing speech enhancement models.

The proposed S-SEGAN DSE model is tested against the existing models F-CSSA–SEGAN^31^, SEGAN^30^, FD-AMS^66^, deep learning^67^ and DNN + AdMBSS^68^ using the PESQ and RMSE metrics in a challenging scenario with 20 dB wind noise, as shown in Figs. 9 and 10. This specific noise level is selected due to its close resemblance to the background noise characteristics commonly encountered in dysarthric speech recordings. Compared to the DNN + AdMBSS model, which has a PESQ of 1.94 and an RMSE of 0.049, the SepFormer-SEGAN model significantly outperforms the other models, with a PESQ of 3.24 and a much lower RMSE of 0.023, indicating better perceived speech quality and greater accuracy. Similarly, compared with the deep learning model, which achieves a PESQ of 1.87 and an RMSE of 0.052, the SepFormer-SEGAN model again shows superior performance. The substantial increase in the PESQ and decrease in the RMSE underscore its effectiveness in enhancing speech quality and reducing error. Compared to the FD-AMS model, which has a PESQ of 2.89 and an RMSE of 0.034, the SepFormer-SEGAN model still has a higher PESQ of 3.24 and a lower RMSE of 0.023, reflecting improved speech clarity and transmission accuracy. Compared with the SEGAN model, which records a PESQ of 3.07 and an RMSE of 0.029, the proposed SepFormer-SEGAN model further improves the PESQ to 3.24 and reduces the RMSE to 0.023, demonstrating enhanced performance in terms of speech quality and error reduction. Finally, compared with the F-CSSA-SEGAN model, which has a PESQ score of 3.12 and an RMSE of 0.026, the SepFormer-SEGAN model shows incremental improvement, with a PESQ of 3.24 and an RMSE of 0.023. This highlights its ability to deliver slightly better perceived speech quality and accuracy. Overall, the SepFormer-SEGAN model outperforms all existing models.

### Evaluation of DSR models

**Fig. 11:**
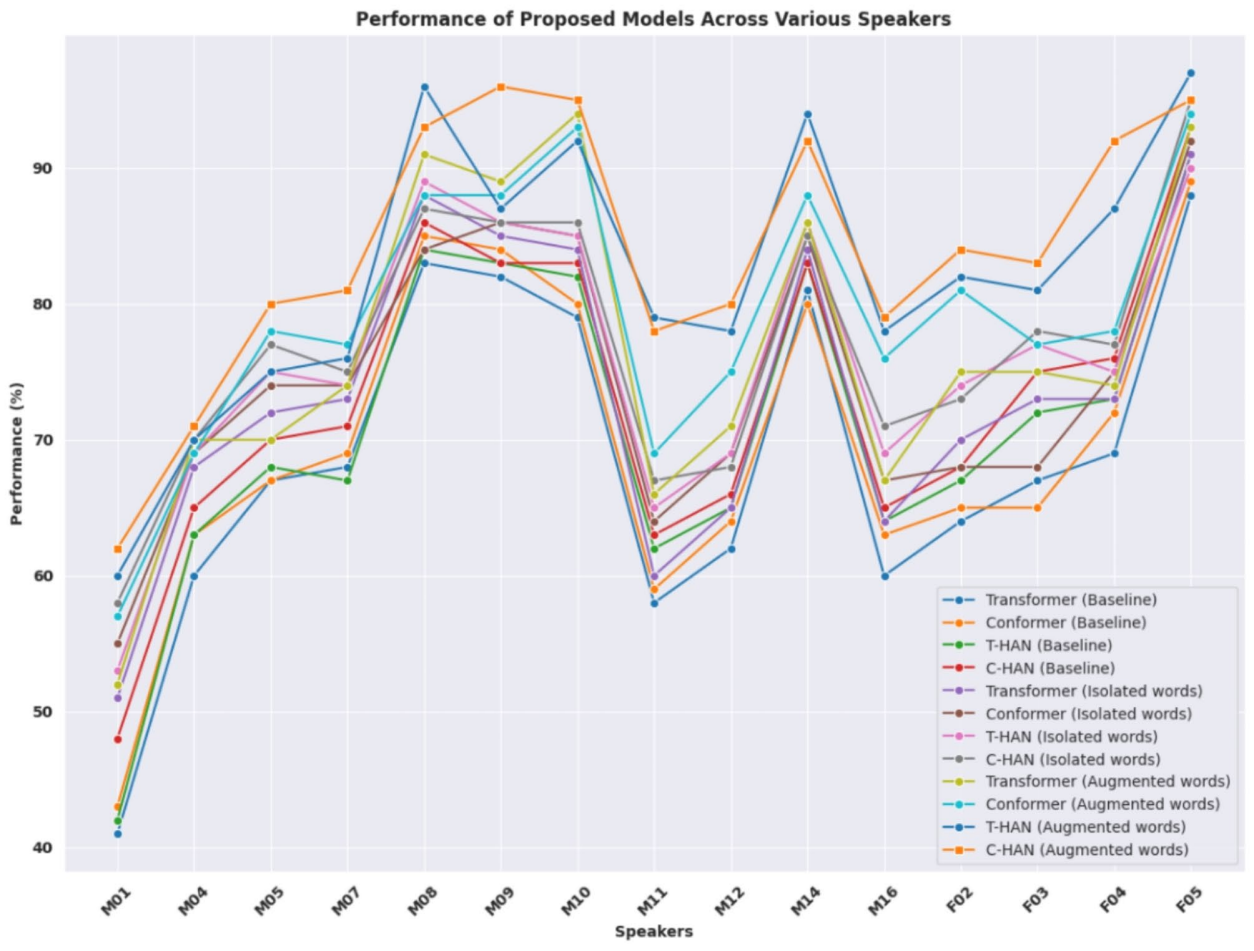
Statistical analysis of the proposed DSR models across different scenarios and speakers.

**Fig. 12:**
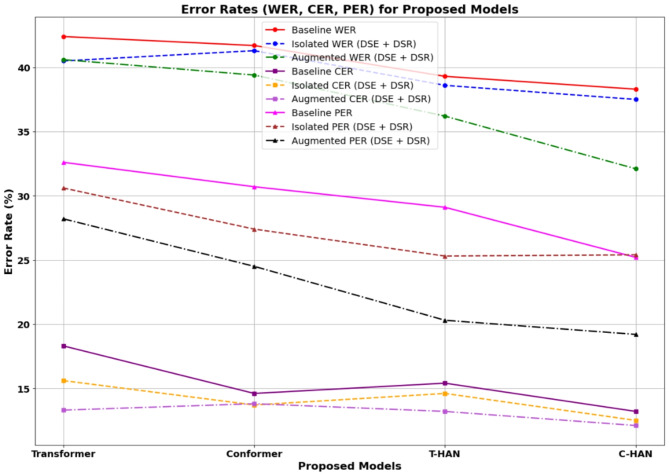
Error rate analysis of the proposed DSR models across different scenarios.

Table 3 offers insights into the performance of the proposed DSR models across different speakers under three distinct scenarios: DS-baseline (isolated words without DSE), isolated words (with DSE + DSR), and augmented words (with DSE + DSR). The models evaluated are the Transformer, Conformer, T-HAN, and C-HAN models. In the DS-baseline scenario, without speech enhancement, the WRA ranges for the Transformer, Conformer, T-HAN, and C-HAN methods are 41–88%, 43–89%, 42–91%, and 48–93%, respectively. C-HAN achieves the highest accuracy of 93% for speaker F05, indicating robust performance even without enhancement. When DSE and DSR are applied to isolated words, the performance improves with the WRA ranges for transformer, transformer, T-HAN, and C-HAN at 51–91%, 55–92%, 53–90%, and 58–95%, respectively, with C-HAN again leading with a maximum accuracy of 95% for speaker F05. Similarly, speakers such as M04 and M07 consistently display moderate performance, with WRAs ranging from 51 to 58% and 42 to 62%, respectively, across all models. In the augmented words scenario, further enhancement is observed with WRA ranges of 52–93%, 57–94%, 60–97%, and 62–95% for Transformer, Conformer, T-HAN, and C-HAN, respectively. T-HAN achieves the highest accuracy of 97% for speaker F05, slightly surpassing C-HAN, which remains highly competitive at 95%. Additionally, in the augmented words scenario, speakers such as M10 and M12 consistently exhibit low performance, with WRAs ranging from 60 to 66% and 62 to 67%, respectively, across all models. In summary, C-HAN emerges as the best model in the DS-Baseline and isolated words scenarios, achieving up to 93% and 95% WRA, respectively, while T-HAN slightly edges out in the augmented words scenario with 97% WRA. However, it is worth noting that C-HAN still performed strongly at 95% WRA in this scenario. The statistical analysis of the proposed models is illustrated in Fig. 11. Table 4 provides a comprehensive analysis of the average WER, CER, and PER across the proposed models under three distinct scenarios, and also includes detailed metrics on precision, recall, and F1 score. In the DS-Baseline scenario, where speech enhancement is not utilized, the models demonstrate varying levels of performance. C-HAN has the lowest average WER of 38.3%, CER of 13.2%, and PER of 25.2%, indicating superior accuracy compared to the other models as shown in Fig. 12. As DSE and DSR are applied to isolated words, there is a notable improvement in performance across all models, with reduced error rates observed. Again, C-HAN maintains its lead with the lowest error rates among all models, with the average WER decreasing to 37.5%, the CER decreasing to 12.5%, and the PER decreasing to 25.4%, suggesting its effectiveness in leveraging speech enhancement techniques. In the augmented words scenario, where DSE and DSR are combined with augmented words, further enhancement in performance is evident. Specifically, the transformer achieves an average WER of 40.6%, CER of 13.3%, and PER of 28.2%. The Conformer achieves a WER of 39.4%, CER of 13.8%, and PER of 24.5%. T-HAN achieves a WER of 36.2%, CER of 13.2%, and PER of 20.3%. C-HAN achieves the lowest error rates, with an average WER of 32.1%, CER of 12.1%, and PER of 19.2%, showing its superior accuracy and robustness. SER analysis underscores the performance of each model, with C-HAN achieving the lowest SER of 29.1% in the DS-baseline scenario, surpassing the Transformer, Conformer, and T-HAN models, which recorded SERs of 34.5%, 32.8%, and 30.4%, respectively. When DSE and DSR are applied to isolated words, the SER decreases across all models, with C-HAN maintaining its lead at 27.2%, followed by T-HAN at 28.3%, Conformer at 29.9%, and Transformer at 31.7%. In the augmented words scenario, further reductions in SER are observed, with C-HAN achieving the lowest SER at 26.7%, closely followed by T-HAN at 29.3%, Conformer at 28.4%, and Transformer at 29.6%. The baseline models show lower precision, recall, and F1 scores, indicating higher error rates. Implementing DSE and DSR with isolated words leads to significant improvements in these metrics. The C-HAN model for augmented words achieves the highest precision at 81.2%, recall at 79.3%, and F1 score at 82.8%, which corresponds with the lowest error rates observed. This confirms that advanced models and techniques contribute to significantly better performance in speech recognition tasks. This consistent demonstration of excellence across all the metrics reinforces C-HAN as the best performing model for processing augmented speech data.

**Table 3:** WRA [%] on the UASpeech dataset under various speaker conditions.

Proposed models	Speakers														
	M01	M04	M05	M07	M08	M09	M10	M11	M12	M14	M16	F02	F03	F04	F05
DS-Baseline- Isolated words (DSR without DSE)															
Transformer	41%	60%	67%	68%	83%	82%	79%	58%	62%	81%	60%	64%	67%	69%	88%
Conformer	43%	63%	67%	69%	85%	84%	80%	59%	64%	80%	63%	65%	65%	72%	89%
T-HAN	42%	63%	68%	67%	84%	83%	82%	62%	65%	83%	64%	67%	72%	73%	91%
C-HAN	48%	65%	70%	71%	86%	83%	83%	63%	66%	83%	65%	68%	75%	76%	93%
Isolated words (DSE + DSR)															
Transformer	51%	68%	72%	73%	88%	85%	84%	60%	65%	84%	64%	70%	73%	73%	91%
Conformer	55%	69%	74%	74%	84%	86%	85%	64%	69%	85%	67%	68%	68%	75%	92%
T-HAN	53%	69%	75%	74%	89%	86%	85%	65%	69%	86%	69%	74%	77%	75%	90%
C-HAN	58%	70%	77%	75%	87%	86%	86%	67%	68%	85%	71%	73%	78%	77%	95%
Augmented words (DSE + DSR)															
Transformer	52%	70%	70%	74%	91%	89%	94%	66%	71%	86%	67%	75%	75%	74%	93%
Conformer	57%	69%	78%	77%	88%	88%	93%	69%	75%	88%	76%	81%	77%	78%	94%
T-HAN	60%	70%	75%	76%	**96%**	87%	92%	**79%**	78%	**94%**	78%	82%	81%	87%	**97%**
C-HAN	**62%**	**71%**	**80%**	**81%**	93%	**96%**	**95%**	78%	**80%**	92%	**79%**	**84%**	**83%**	**92%**	95%

Significant values are in [bold].

**Table 4:** Performance metrics for proposed models across different scenarios: WER, CER, PER, Precision, Recall, and F1 Score.

Proposed models	Avg. WER (%)	Avg. CER (%)	Avg. PER (%)	Avg. SER (%)	Precision	Recall	F1 Score
Baseline-isolated words (DSR without DSE)							
Transformer	42.4	18.3	32.6	35.2	72.5	75.4	68.8
Conformer	41.7	14.6	30.7	34.6	73.0	67.5	70.2
T-HAN	39.3	15.4	29.1	32.8	74.0	68.0	71.0
C-HAN	38.3	13.2	25.2	31.7	75.5	70.0	72.7
Isolated words (DSE + DSR)							
Transformer	40.5	15.6	30.6	33.9	73.8	66.8	69.9
Conformer	41.3	13.7	27.4	34.5	72.0	67.0	71.0
T-HAN	38.6	14.6	25.3	31.5	78.2	70.2	75.2
C-HAN	37.5	12.5	25.4	30.6	76.0	75.6	78.9
Augmented words (DSE + DSR)							
Transformer	40.6	13.3	28.2	33.7	72.8	71.2	70.2
Conformer	39.4	13.8	24.5	32.1	74.2	73.1	76.4
T-HAN	36.2	13.2	20.3	29.3	79.3	78.3	80.2
C-HAN	32.1	12.1	19.2	26.7	81.2	79.3	82.8

#### Comparative study of DSR models

**Fig. 13:**
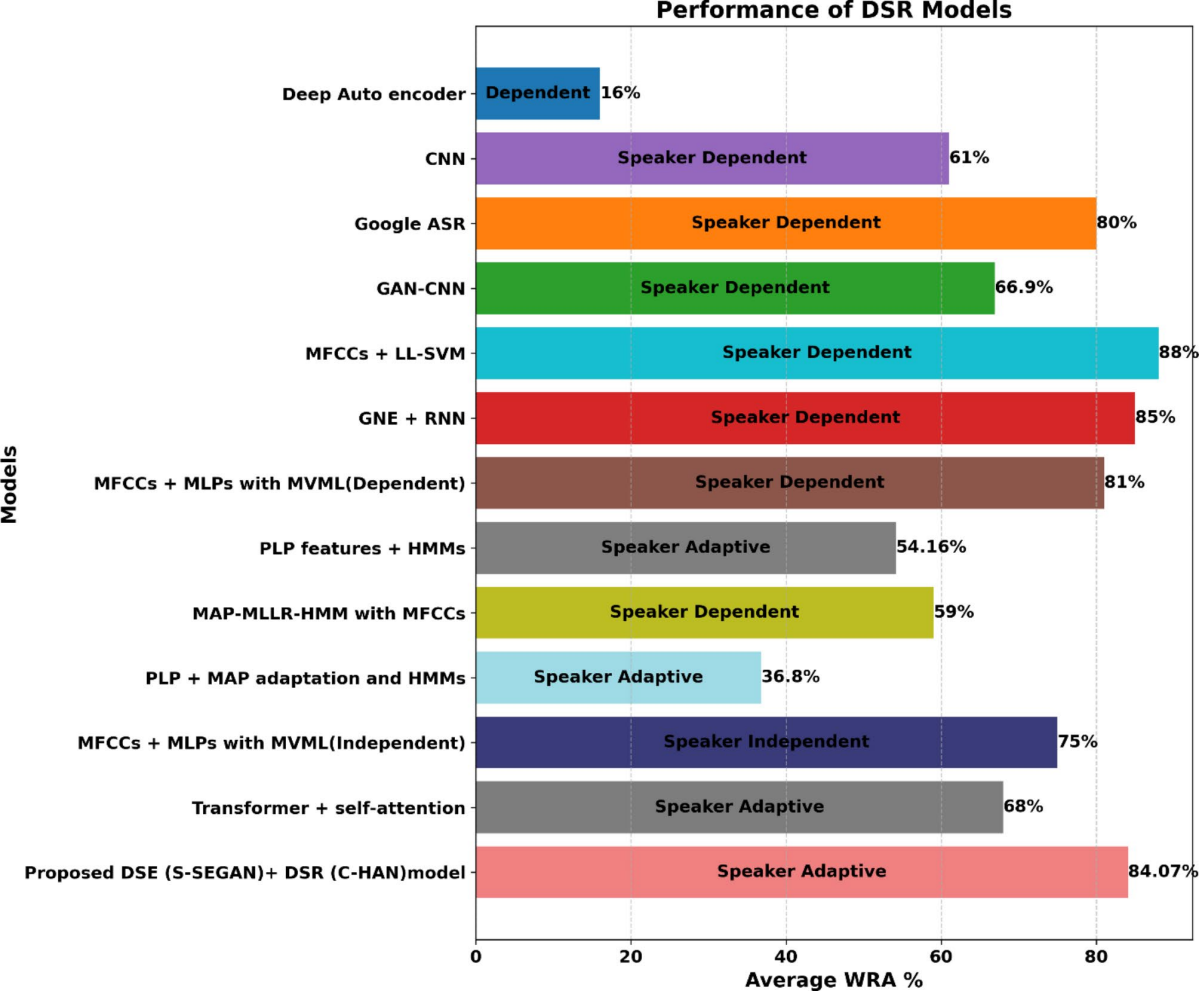
Comparative analysis of DSR models using the UASpeech corpus.

Table 5 displays a comparison between the proposed DSR models and the current state-of-the-art results for the UA-Speech corpus documented in the literature. Previous studies have explored various models, including traditional models such as deep autoencoders^55^ and convolutional neural networks (CNNs)^56^, as well as more advanced approaches such as Google ASR^57^ and GAN-CNNs^4^. In^55^, an evaluation method using bidirectional long short-term memory (BLSTM) neural networks was introduced for objective assessment of dysarthria intelligibility at the sentence level, achieving a WRA of 16%.^56^ employed a 75:25 train/test split strategy across all UA-Speech participants, using dysarthric samples B1 for training and B2 for testing. The results were reported for both original and visually augmented data, focusing solely on real data and achieving a moderate WRA of 61%, with speaker dependency. The highest WRA was documented in^58^, but the method involved mixing and dividing all dysarthric utterances with a 75:25 train/test ratio. This resulted in identical utterances being used for both training and testing due to repeated recordings from various microphone setups, potentially measuring memorization rather than generalization. Moreover, the reported outcomes were based on a limited vocabulary of 29 words. In^59^, the authors categorized each participant’s speech samples into three groups without specifying whether they were based on UA-Speech block categorization (B1, B2, and B3 utterances) or microphone data, potentially encountering similar limitations as^58^. Additionally, their study did not cover all UA-Speech dysarthric speakers, who achieved a WRA of 85%. In^60^, a subsequent WRA of 81% was documented for speaker-dependent systems, while speaker-independent systems achieved a WRA of 75%. However, the study had a small vocabulary size of only 25 words and exclusively utilized speech samples from seven dysarthric participants. Subakan et al.^61^ demonstrated 54.16% accuracy in isolated-word recognition, employing perceptual linear prediction (PLP) features, maximum a posteriori (MAP) adaptation, and hidden Markov models (HMMs) within a speaker-adaptive setup. Surprisingly, the results suggest that dysarthria severity, as measured by the intelligibility rating, may not consistently predict the performance gap between speaker-dependent and speaker-adapted systems. The proposed DSR models underwent rigorous evaluation, as outlined in Table 5. The models were tested on the UA Speech dataset for isolated-word recognition using various architectures and augmentation techniques. The DS-Baseline models, Transformer and Conformer, achieved average WRAs of 68.60% and 69.87%, respectively. The introduction of HAN with the Transformer and Conformer architectures resulted in improved performance, with T-HAN achieving a WRA of 71.07% and C-HAN reaching 73%. Incorporating DSE as a frontend into the DSR models yielded significant performance improvements across all architectures. The Transformer model with DSE + DSR achieved a WRA of 73.40%, while the Conformer model reached 74.33%. The T-HAN and C-HAN models with DSE + DSR showed even more significant improvements, with WRAs of 75.73% and 76.87%, respectively. The augmentation of words further enhanced the performance of the models. The Transformer model with augmented words achieved a WRA of 76.47%, while the WRA of the Conformer model reached 79.20%. Notably, the T-HAN and C-HAN models with DSE + DSR and augmented words demonstrated substantial improvements, with WRAs of 82.13% and 84.07%, respectively. In conclusion, among the proposed models and existing models, C-HAN with DSE + DSR and augmented words achieved the highest WRA of 84.07% under the speaker-adaptive scenario, as shown in Fig. 13. This highlights the effectiveness of the C-HAN architecture combined with DSE and restoration techniques for improving DSR accuracy.

**Table 5:** Comparative assessment of the WRA performance on the UASpeech dataset.

References	Database	Characteristics	Model	Speaker	Performance (Average WRA %)
^69^	UA Speech	Isolated-word	Deep Auto encoder	Speaker Dependent	16%
^70^	UA Speech	Isolated-word	CNN	Speaker Dependent	61%
^71^	UA Speech	Isolated-word	Google ASR	Speaker Dependent	80%
^19^	UA Speech	Isolated-word	GAN-CNN	Speaker Dependent	66.9%
^72^	UA Speech	Isolated-word	MFCCs + LL-SVM	Speaker Dependent	88%
^73^	UA Speech	Isolated-word	GNE + RNN	Speaker Dependent	85%
^74^	UA Speech	Isolated-word	MFCCs + MLPs with MVML	Speaker Dependent	81%
^75^	UA Speech	Isolated-word	PLP features + HMMs	Speaker Adaptive	54.16%
^76^	UA Speech	Isolated-word	MAP- MLLR-HMM with MFCCs	Speaker Dependent	59%
^77^	UA Speech	Isolated-word	PLP + MAP adaptation and HMMs	Speaker Adaptive	36.8%
^74^	UA Speech	Isolated-word	MFCCs + MLPs with MVML	Speaker Independent	75%
^32^	UA Speech	Isolated-word	Transformer + self-attention	Speaker Adaptive	68%
Proposed DSR Models	UA Speech	Isolated-word	Transformer (DS-Baseline)	Speaker Adaptive	68.60%
Proposed DSR Models	UA Speech	Isolated-word	Conformer (DS-Baseline)	Speaker Adaptive	69.87%
Proposed DSR Models	UA Speech	Isolated-word	T-HAN	Speaker Adaptive	71.07%
Proposed DSR Models	UA Speech	Isolated-word	C-HAN	Speaker Adaptive	73%
Proposed DSR Models	UA Speech	Isolated-word	Transformer (DSE + DSR)	Speaker Adaptive	73.40%
Proposed DSR Models	UA Speech	Isolated-word	Conformer (DSE + DSR)	Speaker Adaptive	74.33%
Proposed DSR Models	UA Speech	Isolated-word	T-HAN (DSE + DSR)	Speaker Adaptive	75.73%
Proposed DSR Models	UA Speech	Isolated-word	C-HAN (DSE + DSR)	Speaker Adaptive	76.87%
Proposed DSR Models	UA Speech	Augmented-word	Transformer (DSE + DSR)	Speaker Adaptive	76.47%
Proposed DSR Models	UA Speech	Augmented-word	Conformer (DSE + DSR)	Speaker Adaptive	79.20%
Proposed DSR Models	UA Speech	Augmented-word	T-HAN (DSE + DSR)	Speaker Adaptive	82.13%
Proposed DSR Models	**UA Speech**	**Augmented-word**	**C-HAN (DSE + DSR)**	**Speaker Adaptive**	**84.07%**

Significant values are in [bold].

## Conclusion

The development of ASR systems tailored for dysarthric speech, characterized by impaired articulation, is challenging. Prior research has sought to identify specialized ASRs for dysarthric individuals, surpassing conventional models. However, traditional ASRs, which rely on standard acoustic modeling techniques, are limited in effectiveness due to variability in phoneme production and the scarcity of high-quality dysarthric speech data. This study introduced an innovative approach to significantly enhance DSR accuracy. By integrating SepFormer with the SEGAN, the S-SEGAN model effectively captures long-range dependencies within audio sequences, resulting in enhanced speech signals from dysarthric recordings. SepFormer-SEGAN achieves highest PESQ of 3.24, an RMSE of 0.023, an SNR of 41.98, an STOI of 0.97, and a SegSNR of 17.92. Building upon this enhanced speech, the T-HAN and C-HAN models further enhance DSR accuracy. T-HAN combines the Transformer’s ability to capture long-range dependencies with HAN’s layered attention, effectively prioritizing both the overall context and finer details within dysarthric speech. In the speaker-adaptive scenario, the T-HAN model achieves a recognition accuracy of 75.73% on isolated words from the UA Speech dataset when incorporating DSE. When utilized with S-SEGAN, T-HAN achieves the highest WRA of 97% for speaker F05 using augmented UASpeech. Overall, T-HAN attains an average WRA of 82.13% using DSE and augmented words. C-HAN integrates Conformer’s modeling of short-term and long-term dependencies with HAN’s multilayered attention mechanism, creating a comprehensive and computationally efficient framework. In the speaker-adaptive context, the C-HAN model attains a recognition accuracy of 76.87% on isolated words from the UA Speech dataset through the integration of DSE and DSR. The C-HAN models with DSE and augmented words exhibited the highest average WRAs of 84.07%, with C-HAN achieving the best performance among all the models. This study highlights the effectiveness of combining advanced neural network architectures with DSE and multistage transfer learning of DSR. While the research findings demonstrate significant advancements in DSR, certain limitations remain. One key challenge is the limited availability of large, diverse dysarthric speech datasets, which can restrict the models’ ability to generalize across various speech patterns. Additionally, the complexity of dysarthric speech, marked by high variability in pronunciation, rhythm, and tone, poses ongoing challenges in achieving consistent accuracy across all users. Future research should focus on expanding dysarthric speech datasets and exploring transfer learning techniques to better adapt models to individual speech characteristics. Moreover, investigating more advanced neural architectures and incorporating continuous learning could help improve the models’ adaptability over time, further enhancing real-world applicability and scalability for diverse populations.

## Data Availability

The datasets used for this study are available from the corresponding author upon reasonable request.
